# Epimorphic regeneration in the mammalian tympanic membrane

**DOI:** 10.1038/s41536-023-00332-0

**Published:** 2023-10-18

**Authors:** Sonia M. Scaria, Stacey M. Frumm, Ellee P. Vikram, Sarah A. Easow, Amar H. Sheth, Eliah R. Shamir, Shengyang Kevin Yu, Aaron D. Tward

**Affiliations:** 1 Department of Otolaryngology—Head and Neck Surgery, University of California, San Francisco, CA 94143 USA; 2 Department of Pathology, University of California, San Francisco, San Francisco, CA 94143 USA

**Keywords:** Regeneration, Skin stem cells

## Abstract

Adult mammals are generally believed to have limited ability to regenerate complex tissues and instead, repair wounds by forming scars. In humans and across mammalian species, the tympanic membrane (TM) rapidly repairs perforations without intervention. Using mouse models, we demonstrate that the TM repairs itself through a process that bears many hallmarks of epimorphic regeneration rather than typical wound healing. Following injury, the TM forms a wound epidermis characterized by EGFR ligand expression and signaling. After the expansion of the wound epidermis that emerges from known stem cell regions of the TM, a multi-lineage blastema-like cellular mass is recruited. After two weeks, the tissue architecture of the TM is largely restored, but with disorganized collagen. In the months that follow, the organized and patterned collagen framework of the TM is restored resulting in scar-free repair. Finally, we demonstrate that deletion of *Egfr* in the epidermis results in failure to expand the wound epidermis, recruit the blastema-like cells, and regenerate normal TM structure. This work establishes the TM as a model of mammalian complex tissue regeneration.

## Introduction

Mammals are capable of scar-free injury repair in the embryonic stage; however, after birth, they display increasingly limited capacity to regenerate injured tissues^1^. The characteristic stages of wound healing in adult mammals - (1) hemostasis, (2) inflammation, (3) proliferation, and (4) wound remodeling^2^ - result in tissues that may be functional but do not completely recapitulate the gross morphology and microscopic patterning of the unwounded tissue. In contrast, tetrapods, such as axolotls (*Ambystoma mexicanum)*, are able to regenerate entire complex-tissue limb structures throughout their life-spans^3,4^. The resulting organ resembles the previous unwounded structure in gross morphology, cellular patterning, and full function, with little, if any, remnant of the inflicted injury (scar)^5^. This repair process, termed epimorphic regeneration, is distinguished from mammalian wound healing by a few key features. Epimorphic regeneration is defined by an initial cellular response to wounding prior to the formation of a new part, the formation of a cellularly heterogeneous blastema, and subsequent cellular patterning and morphogenesis resulting in scar-free repair of a tissue or organ^1^. In the axolotl, the site of the wound is rapidly covered by a specialized epidermis, which then serves as a scaffold for blastema formation and the resulting regeneration of the organ^6,7^.

Although mammals are generally believed to lack the ability to regenerate tissues and organs once they develop past the embryonic stage^8^, a number of cases of epimorphic regeneration in adult mammals have been well documented^9–11^. Instances of mammalian regeneration that have been well-documented include ear pinna regeneration of the wild African spiny mouse^1^, rabbit ear pinna regeneration^12^, deer antler regrowth^1^, and distal mammalian digit tip regeneration^13^. Each of these systems share a number of key properties with epimorphic regeneration in non-mammalian species including scar-free repair, blastema formation, and the coordination of developmental signaling pathways between cells in a complex and orchestrated morphogenic process. In each of these examples, these phenomena are generally considered to be observed across mammalian species that retain these organs and thus may represent species specific adaptations acquired more recently in evolutionary history.

The tympanic membrane (TM), or eardrum, is a central component of hearing, relaying sound from the environment to the cochlea^14^. Anatomically, it is separated into two major compartments: the larger but thinner pars tensa (PT), and the smaller but thicker pars flaccida (PF). Both regions are composed of three cellular layers: the external epidermis, the middle fibrous/mesenchymal layer, and the inner mucosal epithelium, which is continuous with the middle ear. In skin elsewhere in the body, the epidermis is stratified, with a basal layer of keratinocytes (KCs) separated from the underlying dermis by a basement membrane. The TM epidermis is a specialized type of skin that is only 3–5 cells thick in humans and 1–3 cells thick in mice and lacks common skin appendages such as hair follicles and sweat glands^15–17^. Moreover, the TM contains an epithelium that is constantly being replaced by turnover occurring every 3 weeks in its homeostatic state^14^. Within the fibrous layer, the TM has two organized layers of collagen fibers, one radial and one circular. Within the PT, the first sound-transducing bone, the malleus, is embedded within this mesenchymal layer, surrounded by nerves and blood vessels. The junction of the PT and the PF as well as the area over the malleus are thought to be the stem/progenitor niche regions of the organ.

In humans, guinea pigs, mice, rats, dogs, and chinchillas, the TM rapidly and spontaneously repairs following injury^18,19^. Although repair of TM perforations is the norm, failure of TM repair in humans is observed clinically and typically associated with chronic infection or severe anatomical trauma^20^. The repair process is so robust that in attempting to create models of chronic TM perforation, investigators have struggled to find animal models where this repair is reproducibly prevented^18,21^. In clinical practice, 750,000 myringotomy procedures are performed in the United States every year, in which a perforation is made in the TM to drain fluid from or relieve pressure in the middle ear space behind the TM^22^. The vast majority of these surgical perforations successfully heal^21^. In order to prevent the healing process and keep the perforation patent, metal or plastic tubes are typically inserted into the TM in order to treat eustachian tube disorders^23^. This robust repair process across adult mammalian species spurred us to characterize the healing of this organ, the cellular and molecular mechanisms of which repair remains poorly understood.

Here, we demonstrate that the mouse TM displays key features of epimorphic regeneration^24^, including the hallmark of scar-free repair. We define a new “wounded” epithelial population of the TM that emerges in response to injury and demonstrate that EGFR is necessary for a robust proliferative response. Taken together, these data provide a cellular roadmap of the TM’s response to injury and categorize its mechanisms as a rare example of mammalian epimorphic regeneration that is observed across species.

## Results

### Injury to the TM induces a robust proliferative response

**Fig. 1:**
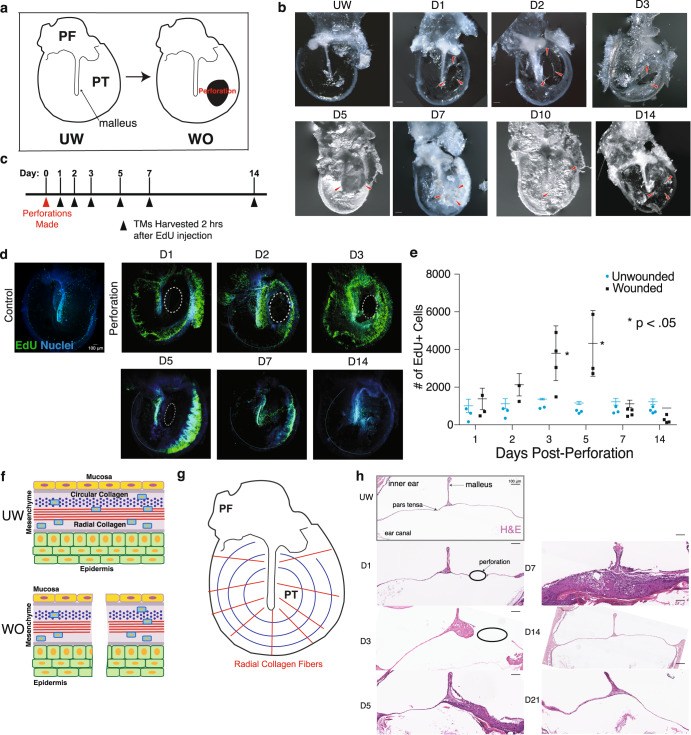
The TM displays a rapid and robust proliferative response to injury macroscopically. **a** Schematic image of a murine TM dissected en bloc to include the pars flaccida (PF) and pars tensa (PT) (left panel). Unwounded (UW) and Wounded (WO) tympanic membranes depicted. Perforations were made in the anterior PT (right panel). **b** Representative wild-type murine TMs harvested from different mice at the indicated timepoints post-perforation. Red arrowheads indicate the site of perforation and gross resolution over time. **c** To characterize the proliferative response to injury, perforations were created in the left TMs of mice on day 0. On days 1, 2, 3, 7, and 14, EdU was injected IP 2 h prior to TM harvest. **d** Edu-labeled whole-mount TMs demonstrate a peak proliferative response 3 days post-injury, with resolution by day 14. White dashed circles indicate the perforation. **e** Graph of the number of EdU+ cells in a 400 × 1200 μm area over the malleus in response to injury. Results of *t*-tests for WO vs. UW TMs at a single time-point are indicated with the black bars with the outer limits representing standard deviation; *p < 0.05. **f** A cross-sectional view of the TM demonstrates the 3 major layers: inner mucosa, middle mesenchyme, and outer epidermis. When wounded, the injury crosses all 3 layers. **g** The mesenchymal layer contains two layers of inner circular and outer radial collagen fibers. **h** Hematoxylin and Eosin (H&E) stained sections of the TM at the level of the perforation mid pars tensa at multiple timepoints following perforation. Tissue stratification peaks at Day 7 and resolves by Day 21. The epidermal layer is oriented downward, and the black circle indicates the site of the perforation. Scale bars: (**b**) 200 μm (**d**): 100 μm (**h**) 100 μm.

To characterize the TM response to injury, we first assessed gross tissue morphology during the time course of wound repair. We perforated the left TMs of mice in the postero-inferior quadrant of the pars tensa, using the right TMs as unwounded controls (Fig. 1a). We harvested TMs at multiple timepoints during wound healing and imaged whole mounts (Fig. 1b). Macroscopically, there is a large buildup of tissue with thickening of the TM by day 3 post-perforation. This increase in tissue volume persists through day 7. By day 14, the tissue had drastically remodeled and decreased in volume back to the gross appearance of an unwounded TM, without apparent evidence of the prior injury. Across all experiments, we found that TM perforations observed on day 7 or later (240/240) were closed.

We next sought to characterize the spatial and temporal proliferative response following TM injury. We created perforations as previously described and pulsed the mice with EdU two hours prior to harvesting the TMs (Fig. 1c). Within 18 h of perforation, there was a detectable increase in proliferation (*p* = 0.0082) (Supplementary Fig. 1a, b). This early increase in EdU-labeled cells is faster than that seen in mammalian wound healing in the skin, which typically shows an increase in proliferation 48 h following wounding^25^. At one day post-injury, there was a marked increase in proliferation in the same regions that are proliferative under homeostasis: the tissue over the malleus, the junction of the pars tensa and pars flaccida, and near the annulus^14^ (Fig. 1d). By day 5, we observed the peak in proliferation, with a 5.5-fold increase in EdU+ cells over the whole TM (*p* = 0.0043) (Fig. 1e). By day 7, the proliferation had substantially decreased, and by day 14, the proliferation pattern resembled that of the unwounded TM.

The proliferative response to wounding in the TM appeared to involve the whole organ rather than just the area around the wound site. Homeostatic turnover of the entire TM epidermis takes approximately 3 weeks^14^. To determine how the rate and location of turnover are impacted by wounding, we labeled the TM KCs with EdU supplied via drinking water, then removed the source of EdU upon injury of the TMs and harvested the tissues over the course of four weeks (Supplementary Fig. 1c). Because the proliferating cells in the TM under homeostatic conditions are almost exclusively KCs, the majority of cells labeled with this protocol are KCs. We observed the loss of the EdU label in the TM epidermis broadly by day 7 rather than the typical 3 weeks seen in the homeostatic TM (Supplementary Fig. 1d). Thus, the robust proliferative response to wounding of the TM accelerates some combination of cell loss, migration, and/or proliferation, of the TM epidermis throughout the organ.

### TM perforation repair restores all layers of the TM

**Fig. 2:**
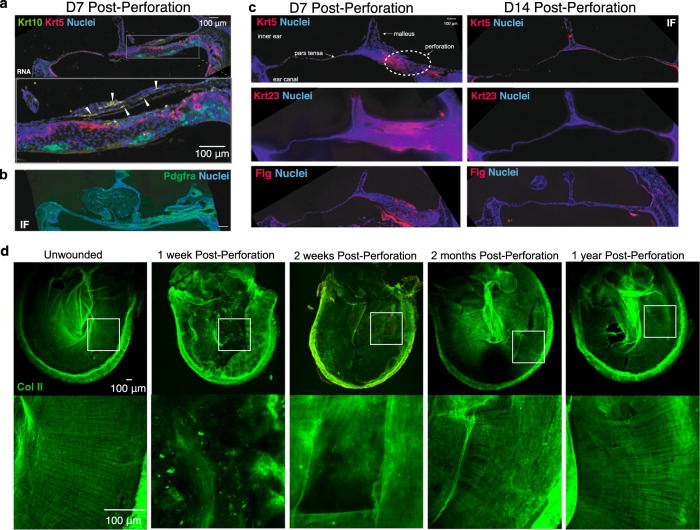
The TM regenerates without permanent scarring. **a** RNAscope showing expression of *Krt5* (red) and *Krt10* (green) in a cross-section of the TM from day 7 post-injury, illustrating transient mixed stratification within the epidermis. The yellow magnified region highlights an area directly over the wound site. White arrows denote cells co-expressing *Krt5* and *Krt10*. **b** Immunofluorescence (IF) for Pdgfra in a TM cross-section from day 7, demonstrating the newly formed multi-lineage stratification of the TM. **c** IF for Krt5, Krt23, and Flg in TM cross-sections from day 7 and day 14 post-injury, demonstrating expansion and quick dissipation of cells positive for these markers. **d** IF for COLII (green) in representative whole-mount TMs harvested at the indicated timepoints post-injury reveals restoration of normal collagen patterning of the TM by year 1 (*n* = 5 mice per timepoint, except *n* = 3 for 1 year). Scale bars: 100 μm.

Most skin wounds heal by filling a defect that is bound by tissue on three sides and results in incomplete reconstitution of pre-existing skin structures^26^. In contrast, wounding of the TM creates a perforation that goes through epidermal, mesenchymal, and mucosal layers (Fig. 1f) and is bound only by tissue along its edges. It also disrupts the organized layers of radial and circular collagen fibers in the TM (Fig. 1f, g). We examined H&E-stained cross-sections of the TM at the area of injury over time (Fig. 1h). At one day post-perforation, we began to see some expansion and stratification of the keratinocytes over the malleus and annulus. By day 3, this population had massively expanded, and the TM had thickened substantially. By day 5, the perforation was typically filled by a mass of cells (~16× thicker than the UW TM) including keratinocytes (Fig. 2a), Pdgfra+ mesenchymal cells (Fig. 2b), and immune cells. At day 7, the TM reached its maximal thickness (~52× thicker than UW), with marked expansion of the epidermis that extended beyond the site of perforation. We observed keratinization on the epidermis side of the TM with sloughing of keratin debris at day 7 while UW TMs do not usually display keratinization within the pars tensa. On day 14 post-injury, the TM had restored its original thickness and appeared histomorphologically identical to the unwounded TM, with resolution of the multi-lineage disorganized cell mass and intact mucosal, connective tissue, and epidermal layers. This process resembles the steps of epimorphic regeneration in the axolotl limb, including expansion of a wound epidermis, formation of a multi-lineage blastema-like structure, and subsequent morphogenesis and resolution^1^.

We next investigated the differentiation state of the KCs during the time course of healing. In the initial phases, there is an expansion of nucleated, basal-appearing Keratin5+ (K5 + ) keratinocytes. At later timepoints, we observed heterogeneous labeling for markers of basal KCs (K5), differentiated KCs (Keratin10, Keratin23, Filaggrin^27^), and mesenchymal cells (Pdgfra) throughout the thickness of the TM by in situ hybridization (ISH) (Fig. 2a) and immunofluorescence (IF) (Fig. 2b, c). We observed cells positive for both K5 and Keratin10 (Fig. 2a). This disorganized stratification and differentiation was most prominent at day 7 post-injury and resolved by day 14.

We next asked how the tissue remodeled from such a great thickness at day 7 to the TM’s original thickness by day 14. Using TUNEL-staining to detect apoptosis, there was a mild increase in apoptosis at day 8 and day 10, but not significantly enough to account for the massive thinning of the tissue (Supplementary Fig. 2a). However, a large cornified crust likely composed of sloughed and differentiated KCs was observed overlying the regenerated region^28^. Taken together with the histological features, we conclude that the majority of tissue mass reduction is likely caused by differentiation of KCs and subsequent keratinization, and formation a crust, which is later lost in the external auditory canal, consistent with previous observations in humans and other mammals^21^.

### The TM displays scar-free repair

Repair of other mammalian epithelial tissues such as skin generally results in scar formation^29^, defined as the deposition of fibrotic tissue that does not fully recapitulate the original tissue structure and function. We next sought to determine whether repair of the injured TM similarly resulted in a scar by studying the characteristic organization of collagen fibers in the TM (Fig. 1g). The middle mesenchymal layer of the TM is primarily composed of Collagen II (COLII) during homeostasis, with the appearance of Collagen I (COLI) being associated with injury^30^. We performed IF for COLII and COLI in the unwounded TM and at multiple timepoints post-injury to visualize the collagen patterning of the injured TM (Fig. 2d and Supplementary Fig. 2b). At 2 weeks following perforation, the TM, though grossly healed, did not display the typical circular and radial COLII fiber patterning typical of an unwounded TM, and a small amount of COLI was present localized to the wound site. However, by 2 months, the COLII patterning appeared nearly restored to the pre-injury state (*n* = 15 for COLII), and COLI was minimally present at the site of perforation (*n* = 15). At one year following injury, there was no detectable difference from homeostasis in the appearance of the COLII radial and circular fiber structure (*n* = 3). Thus, the healed TM restores its cellular structure and its highly organized connective tissue layer without evidence of a scar.

### TM cell populations undergo transcriptional shifts during healing

**Fig. 3:**
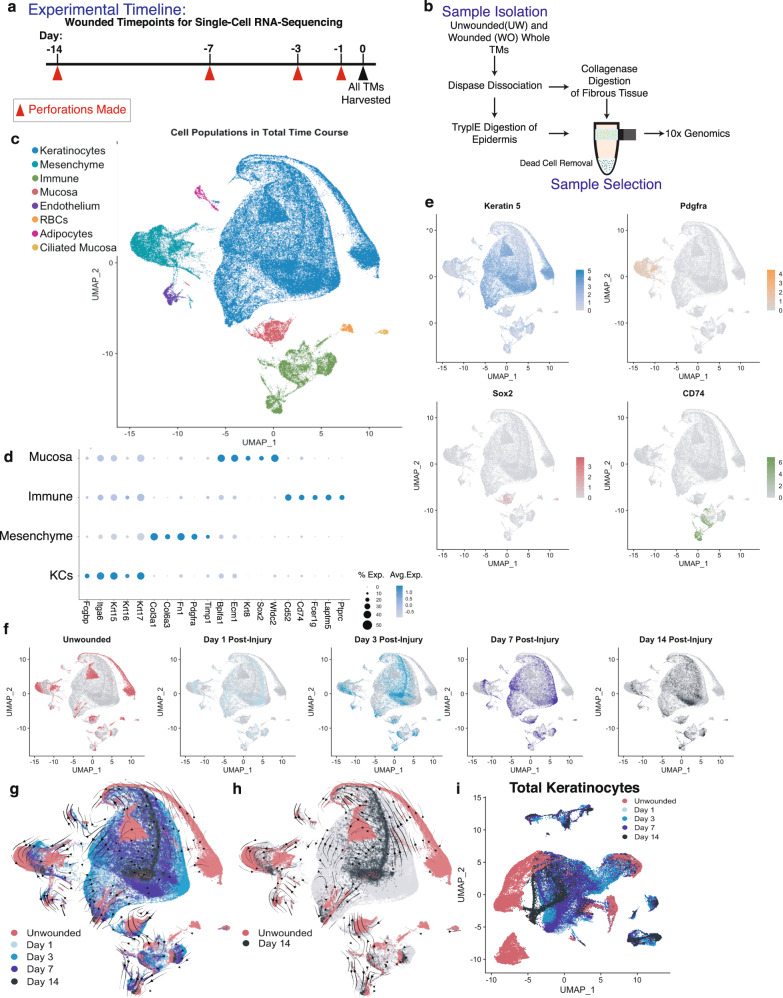
Single-cell RNA sequencing (scRNA-seq) reveals the transcriptional shifts of the regenerating TM. **a** TMs at various timepoints post-perforation were harvested and processed at the same timepoint. **b** Single cells were isolated from unwounded and wounded TMs for scRNA-seq. **c** UMAP visualization of all cell clusters in the merged scRNA-seq data, including wounded and unwounded states, compiled and analyzed by Seurat. **e** UMAP plots showing expression of *Krt5* in the KCs, *Pdgfra* in mesenchymal cells, *Sox2* in mucosal cells, and *Cd74* in immune cells. **d** Dot Plot highlighting top marker genes of the KC, mesenchymal, mucosal and immune cell populations. **f** UMAP plots highlighting the cells based on which unwounded/wounded timepoint they originated from. **g**, **h** Separate UMAP visualizations of all of the unwounded and wounded cells with RNA velocity vectors super-imposed, calculated using the scVelo package with all cells highlighted (**g**) and with only the unwounded and day 14 cells highlighted (**h**). **i** Separate UMAP visualization of KCs from all timepoints, which were re-clustered independent of other cell types. Cells are highlighted based on their original injury timepoint.

We next sought to generate a global transcriptional map of cells during TM perforation repair. We performed single-cell RNA sequencing using the 10× Genomics Single Cell Solution v3 platform on dissociated TM tissues at 4 different timepoints post-injury and compared their transcriptional landscape to that of unwounded controls (Fig. 3a, b). We selected timepoints at 1, 3, 7, and 14 days after injury based on gross and microscopic changes observed in our prior experiments that indicated that these were key timepoints of wound epidermis response, robust proliferative expansion of cell layers, and morphologic resolution.

Datasets for each timepoint were analyzed with Seurat v3^31^ and the results visualized two-dimensionally using uniform manifold approximation and projection (UMAP) (Supplementary Fig. 3). As we were interested in transcriptional changes over the time-course of repair, we merged the 5 datasets and reanalyzed the combined object with Seurat (Fig. 3c–e). Eight major cell types were identified by their marker gene expression: keratinocytes, non-ciliated mucosa, ciliated mucosa, mesenchyme, immune, red blood cells, adipocytes, and endothelium^32,33^. In order to identify distinct subpopulations of cells along the time course, we used CellFindR^34^, an algorithm that incorporates unbiased iterative sub-clustering to identify biologically significant populations. This revealed multiple distinct subpopulations of cells, many of which were specific to distinct stages of TM healing. When the cells were colored by the original timepoint in the merged UMAP, cells within each time point occupied a distinct transcriptional space, indicating that they were dissimilar from cells at other timepoints within the major cell populations of the TM (Fig. 3f). Though the TM tissue structure appeared grossly resolved by day 14 (Fig. 1b), the transcriptional states of the cells did not fully restore to the unwounded state in this time frame. Nevertheless, the general trend over the time course suggested that the cells’ transcriptional states were continuing to become increasingly similar to the unwounded state based on the spatial locations of the cells from each timepoint on the merged UMAP and RNA velocity vector analysis (Fig. 3g, h). When tracking the velocity vectors of the cells throughout the time course, we see that cells move outwards in the transcriptional space until the maximal distance at day 3; the arrows then reverse and move back towards the center of the UMAP, approaching but not overlapping with the unwounded cells by day 14 (Fig. 3h), suggesting that the cells’ transcriptional signature may become increasingly similar to the unwounded state at these later timepoints.

### Single-cell analysis reveals that distinct layers of the TM demonstrate time-dependent transcriptional shifts and turnover following injury

A key feature of epimorphic regeneration is layer-specific and multi-lineage replacement of tissue. Therefore, we sought to investigate the transcriptional shifts over time within each layer of the TM during repair. To separately investigate each layer of the TM, we subclustered the merged dataset into specific cell types: keratinocytes, mesenchymal cells, immune cells, and mucosal cells (Fig. 3i, Supplementary Fig. 4).

Keratinocytes make a large transcriptional shift almost immediately after perforation; at day 1 after wounding, they occupy an almost entirely separate transcriptional space from that of unwounded keratinocytes (Fig. 3i). There are changes in expression of numerous transcripts, such as *Lgals1, Cald1, BC100530, Odc1*, and *Gpr15L*, some of the highest differentially expressed genes, as well as an increase in expression of proliferation-associated genes like *Mki67* and *Ccnd1* in the keratinocytes. This is in line with the rapid proliferative response we observe as early as 18 h after perforation (Fig. 1d, Supplementary Fig. 1b). In contrast, the other layers of the TM display a more delayed transition transcriptionally, consistent with associated histologically apparent changes (Fig. 2) and prior immunostaining data^35^. In homeostasis, very few cycling cells are seen in the layers of the TM outside of the epidermal layer^14^. However, during TM repair, multiple cell types, including mesenchymal, mucosal, and macrophage populations, express proliferation markers including *Mki67*, *Top2a*, and *Ccnd1*, consistent with a multi-lineage response to injury (Supplementary Fig. 4a–d). We confirmed the timing of increased proliferation in the keratinocyte, mucosal, and mesenchymal layers after injury by injecting mice with EdU 2 h prior to harvesting TMs and co-staining with Krt5 (Supplementary Fig. 4e), Sox2 (Supplementary Fig. 4f), or Pdgfra (Supplementary Fig. 4g) antibodies, respectively, to identify cells actively proliferating at the selected points in time. Krt5+ cells showed an increase in proliferation (EdU labeling) at one day post-perforation, while Sox2+and Pdgfra+ cells showed an increase at 3 days post-perforation. Moreover, the Sox2+ cells co-stain with Edu primarily over the wound site while the Pdgfra+ cells co-stain primarily over the malleus at day 3, indicating that the pattern of regeneration and migration for the mesenchymal cells is more closely in line with that of the keratinocytes, but the mucosal cells likely do not show this same wounded migratory phenotype.

To better understand the populations of mesenchymal cells present during TM wound healing, we computationally isolated the mesenchymal cells from the merged dataset and re-clustered them using CellFindR. We discovered 15 subclusters (Supplementary Fig. 5a, b), including several subpopulations specific to the wounded state. Labeling by timepoint revealed a large transcriptional shift at day 3 following injury (Supplementary Fig. 5c), with differential expression of multiple genes, including upregulation of *Mt1, Mt2*, and *Timp1* amongst the highest expressed genes (Supplementary Fig. 5d). Cells in the unwounded state displayed higher expression of canonical mesenchymal markers, such as *Vim*, *Pdgfra*, *Fn1*, *Col1a1,* and *Col1a2* (Supplementary Fig. 5e–i)^36,37^. In contrast, in the wounded state, there appeared to be activation and proliferation of an *Acta2+* (cluster 1.1.2) subpopulation as well as a distinct *Coch+* population (cluster 1.1.4), both of which were detectable as early as day 1 following perforation. RNA velocity analysis revealed transcriptional trajectories that transitioned through wounded states and ultimately returned to a more unwounded like state (Supplementary Fig. 5m–o). By day 14, the cells are still distinctly different than the wounded state, but the cells show directional RNA movement to the unwounded state (Supplementary Fig. 5o). To corroborate our day 1 findings with our initial analysis that revealed the largest transcriptional shift at day 3 post-injury, we examined the shifts in UMAP space from the unwounded state to day 1 specifically and found that the day 1 wounded and unwounded pars flaccida cells were closely overlapping, indicating that the transcriptional identities immediately post-injury of the mesenchyme still largely resemble that of the unwounded state; more significant transcriptional shifts were thus occurring at later timepoints (Supplementary Fig. 5p, q). Notably, the unwounded pars tensa cells do not overlap with the day 1 wounded cells, which suggests a transcriptional shift within these cells within 24 h of injury. ISH of TM sections revealed Coch+ mesenchymal cells within the blastema of the healing TM (Supplementary Fig. 5r). Thus, the mesenchyme displays a robust response to wounding, coinciding with blastema formation, but following the induction and expansion of the wound epidermis.

Among immune cells, there are several populations detectable in both the wounded and unwounded states. We identified macrophages, dendritic cells, B cells, T cells, Langerhans Cells, myeloid-derived cells, and monocytes (Supplementary Fig. 6a, b). Within these populations, the transcriptomes showed clear differentiators between the wounded and unwounded state with distinct clusters of macrophages, myeloid derived suppressor cells, and T-cells appearing at distinct timepoints after wounding (Supplementary Fig. 6b, c). Canonical Markers of the major immune populations were used to identify these sub-clusters (Supplementary Fig. 6d–h). In examining the UMAP of immune cells labeled by timepoint, the largest transcriptional changes arise between days 1 and 3 post-injury (Supplementary Fig. 6c). ISH for *Cd68*, a marker of macrophages and monocytes^38^, validated these cell populations and revealed an increase over the malleus at day 7 post-injury (Supplementary Fig. 6i).

Finally, we examined mucosal cells for distinct subpopulations and transcriptional changes in response to injury. Although we identified distinct ciliated and non-ciliated mucosal cells, we did not identify any populations specific to the wounded state.

### Injury leads to the development of a specialized wound epidermis

**Fig. 4:**
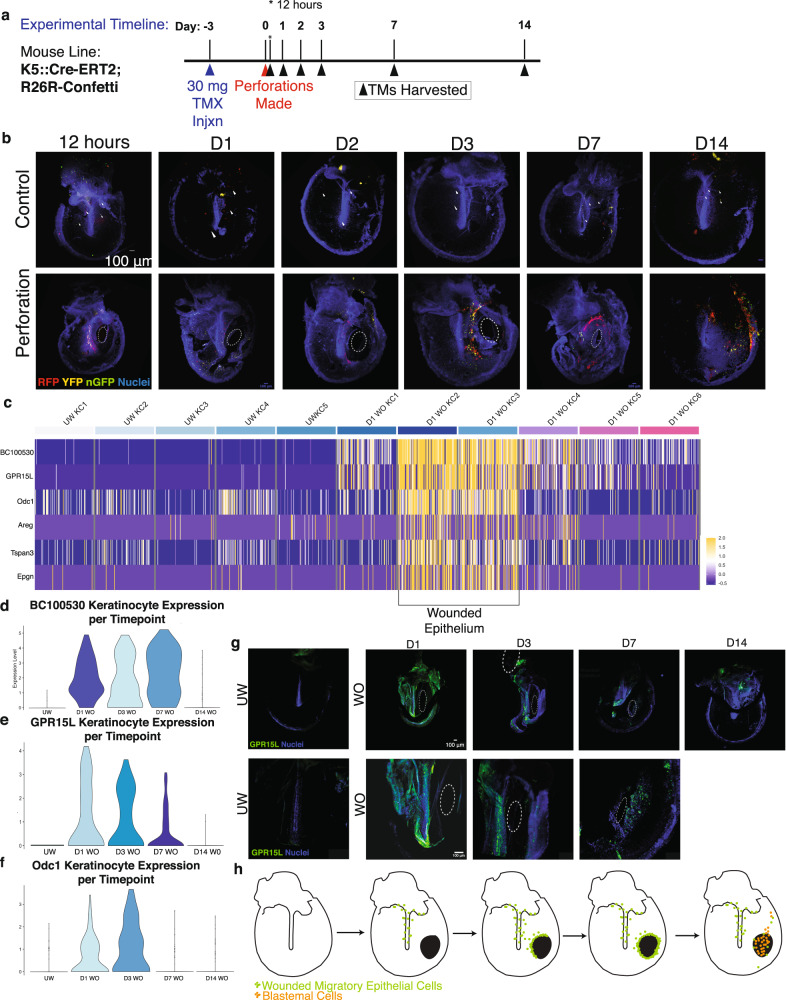
A migratory wounded epithelium forms within one day post-injury. **a** *Krt5-CreERT2;R26R-Confetti* mice were injected with a single dose of 30 mg of tamoxifen 3 days prior to TM injury to induce minimal labeling of TM KCs. Perforations were made in the left TMs of mice on day 0, and both TMs were harvested at the indicated timepoints. **b** Control TMs from the right ear (top row) show sparse labeling with the Confetti reporter. Perforated TMs from the left ear (bottom row) show increased labeling over the malleus at early timepoints, with a concentration of labeled cells at the site of injury at later timepoints (*n* = 5 mice per timepoint). White dotted circles indicate the perforation **c** Heat-map showing expression of top genes associated with the wounded epithelial cell state in KC clusters from the unwounded TM and day 1 post-injury. Each column represents a single cell. **d**–**f** Violin Plots showing expression of *BC100*530 (**d**), *Gpr15L* (**e**), and *Odc1* (**f**) in KCs from each timepoint. **g** RNAscope for *Gpr15L* (green) expression on whole-mount TMs throughout the regenerative time course (top row). Higher magnification regions show expression of *Gpr15L* (green) over the malleus at day 1 and near the perforation by day 7 (bottom row), suggesting migration of the wounded epithelium. Scale bars: 100 μm (H) Cartoon model of the origination and migration of the wounded epithelium on the TM.

We next sought to more deeply characterize the initial response of keratinocytes following injury. We investigated whether the cells responding to injury were the progeny of a pre-existing K5+ population of progenitor cells in the TM, present in the TM under homeostasis^14^. *Krt5-CreERT2;R26R-Confetti* mice were administered a single dose of 30 mg of tamoxifen to label a minimal subset of keratinocytes, including, those cells capable of acting as progenitors in an injured state. We optimized the dosage of tamoxifen to capture a small fraction of cells within the overall widespread K5+ epithelial population on the TM. The subsequent clones that expanded after perforation, expressing YFP, CFP, RFP, or GFP, should represent the progeny of resident stem and/or progenitor cells that rapidly expand in response to injury. The TMs were injured 3 days later and harvested at various timepoints to visualize the K5+ daughter cells (Fig. 4a). In unwounded TMs, we observed rare K5+ cells, with little evidence of subsequent expansion or migration. In contrast, in wounded TMs, we observed an expansion of K5+ lineage-traced cells as early as 12 h following perforation, predominantly over the malleus and at the junction of the PF and PT (Fig. 4b). On days 1–7, there was further expansion of K5+ lineage-traced cells, and their localization shifted to the site of the wound (Supplementary Fig. 7a, b). This is consistent with the rapid expansion of a K5+ population in the stem/progenitor niches of the TM within 24 h post-injury, with subsequent migration to the site of injury.

Given the rapid proliferative and migratory responses we observed, we next sought to characterize the transcriptional events within the keratinocyte populations. We first investigated the keratinocyte shifts occurring on the first day post-injury by merging and re-clustering the unwounded and day 1 datasets. We found that keratinocytes generally expressed a distinct transcriptional program by one day following wounding (Supplementary Fig. 7c). Furthermore, we identified a subpopulation of keratinocytes that expressed genes distinct from any seen during homeostasis (Figs. 4c, Supplementary Fig. 7d, e). We hereafter refer to this transient population as the “wounded epithelium”. In contrast, the other keratinocyte populations had high differential expression of genes that more clearly aligned with markers seen at homeostasis, such as *Fam213a*, *Fcgbp, Dst*, and *Apoe*, and likely represent less radically transcriptionally shifted versions of these populations. The wounded epithelium’s highest differentially expressed transcripts were for genes not expressed in unwounded keratinocytes, such as *BC100530*, *Odc1*, and *Gpr15L* (Fig. 4c). There were 167 genes that separated this population from all other keratinocyte populations at day 1, when filtered for at least a two-fold change in average differential expression. This wounded epithelium persisted through day 7 but had largely dissipated by day 14, with top markers of the wounded epithelium no longer detectable (Fig. 4d, e). We used ISH for *Gpr15L*, one of the top three differentially expressed genes in this population, to identify the spatial localization of this population through the time course of repair (Fig. 4g). Cells positive for *Gpr15L* first appeared over the malleus on day 1 post-injury and along the annulus and then around the wound site at later timepoints (Fig. 4g, h). This localization is consistent with the expansion and migration of the K5+ lineage-traced population that we had observed earlier.

### The wounded epithelium activates EGFR signaling

**Fig. 5:**
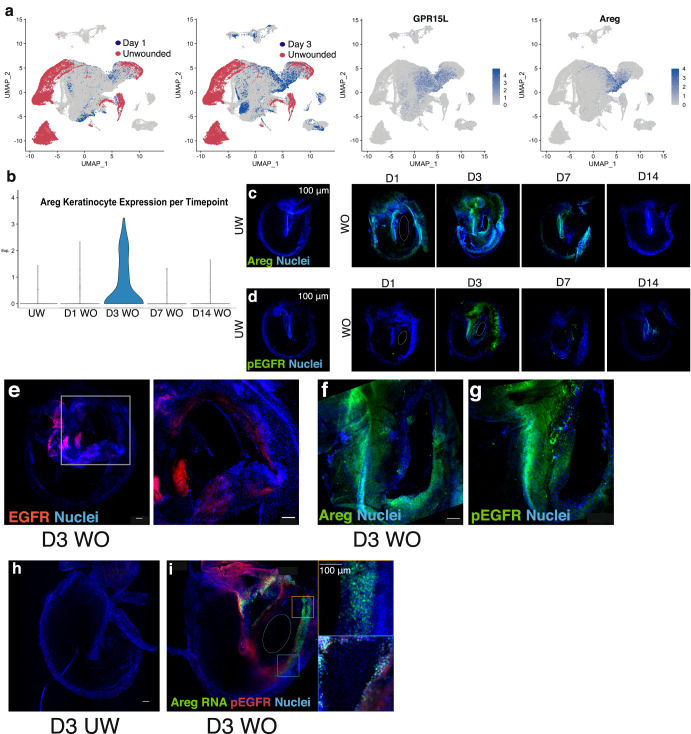
A blastema-like structure with activation of the EGFR pathway arises at the site of injury. **a** UMAP visualization of the KCs from all timepoints with the unwounded cells highlighted in red and day 1 or day 3 KCs in blue. The right panels highlight cells with the highest expression of *Gpr15L* or *Areg*. **b** Violin plot showing expression of *Areg* in KCs from each timepoint. **c**, **d** IF for Areg (**c**) and pEGFR (**d**) in unwounded and wounded whole-mount TMs shows peak expression of both at day 3 post-injury. White dotted circles indicate the perforations (**e**–**g**) IF for EGFR (**e**), Areg (**f**), and pEGFR (**g**) on representative whole-mount TMs from the day 3 wounded state. Higher magnification shows increased staining around the site of perforation (**h**, **i**) IF for pEGFR co-stained with *Areg* RNA using RNAscope on a whole-mount TM of the day 3 unwounded(H) and wounded(I) state. Panels (I’) and (I”) show zoomed-in images of co-localization of *Areg* and pEgfr from the D3 WO TM. Scale bars: 100 μm.

Among the top upregulated genes within the wounded population were *Amphiregulin* (*Areg*), *Epigen* (*Epgn*), *Heparin-binding Egf* (*Hbegf*), *Epiregulin* (*Ereg*), and *Tgfa*, all ligands of EGFR, which has an established role in wound healing involving KCs^39^ (Fig. 4c). *Areg* expression appeared to closely overlap with *Gpr15L* expression in the KC populations (Fig. 5a). Thus, we hypothesized that the wounded epithelium may be activating signaling through the EGFR pathway and that activation of this pathway may be required for TM regeneration.

To test whether the increase in *Areg* expression was specific to the post-injury state of the TM, we queried the merged dataset of keratinocytes from all timepoints. We found that *Areg* was maximally expressed in day 3 keratinocytes (Fig. 5b). Using IF, we observed that Areg was present in keratinocytes from day 1 to day 7, initially localized to the malleus and shifting to keratinocytes around the wound site at day 3 (Fig. 5c). This expression pattern was consistent with levels and localization of *Areg* mRNA detected via ISH (Supplementary Fig. 8a–c). This spatial and temporal distribution matched that seen previously in the wounded epithelium (Figs. 4b, h).

We next sought to decipher whether *Areg* was acting in an EGFR-independent^40^ or dependent fashion, as both mechanisms had been reported to drive keratinocyte proliferation. Using IF, we stained for pEGFR at various timepoints post-injury and found a robust increase in pEGFR signal (Fig. 5d). The spatial localization of pEGFR and Areg was consistent with the pattern of EGFR expression over the malleus and at the wound site on day 3 (Fig. 5e–g) and corresponded to the localization of *Areg* RNA (Fig. 5h, i). We next co-stained for pEGFR and *Gpr15L* RNA and identified a subset of *Gpr15L+* cells that were positive for pEGFR (Supplementary Fig 8H, I). Thus, EGFR signaling appears to be activated in a ligand-dependent autocrine fashion within the wounded epithelium.

### Deletion *of Egfr* abrogates the TM’s rapid proliferative response to injury

**Fig. 6:**
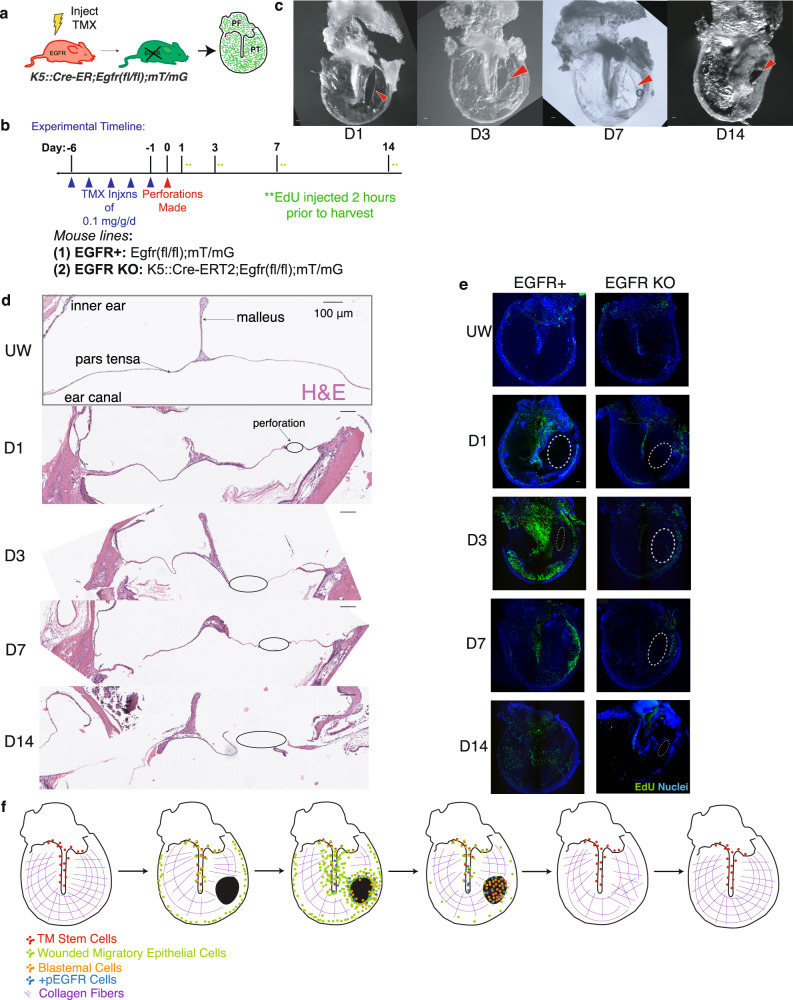
Egfr is required for the early TM regenerative response. **a** *EGFR* deletion was induced in vivo in adult *K5-CreERT2; Egfr*^*fl/fl*^*; R26*^*mTmG/mTmG*^ mice via tamoxifen injection. *Egfr*^*fl/fl*^*; R26*^*mTmG/mTmG*^ mice served as negative controls. **b** Tamoxifen was administered for five consecutive days to induce complete recombination in TM keratinocytes prior to perforation. TMs were then harvested at multiple timepoints post-injury, with EdU injection 2 h prior to each harvest. **c** Wounded TMs isolated from different EGFR KO mice at multiple timepoints post-injury demonstrate incomplete resolution of the injury. Red arrowheads indicate the site of perforation. **d** H&E-stained sections of the TM at the level of the perforation mid-pars tensa at multiple timepoints following perforation in EGFR KO mice, showing that tissue stratification and wound closure is absent. The epidermal layer is oriented downward, and the black circle indicates the site of the perforation. **e** EdU labeling in control EGFR+ mice post-perforation (left) demonstrates peak labeling at day 3 and closure of the perforation by day 14. EdU labeling in EGFR KO mice post-perforation (right) shows a lack of a proliferative response and incomplete wound closure. White dashed circles indicate the perforation. **f** Schematic of our proposed model of TM regeneration. Scale bars: 100 μm.

To test whether EGFR signaling was required for TM regeneration, we generated a genetically engineered mouse model that allowed for conditional deletion of *Egfr* in keratinocytes (K5-CreERT2;*Egfr*^*fl/fl*^*:R26*^*mTmG/mTmG*^) (Fig. 6a). We also utilized the *mT/mG* Cre reporter, in which cells heritably convert from tdTomato+ to EGFP+ in response to Cre recombinase expression^41^. With this mouse model, cells in which *Egfr* is deleted will thus express EGFP. We administer tamoxifen for 5 sequential days to induce Cre activity and then perforated TMs 24 to 48 h after the last injection to ensure complete degradation of EGFR protein, which has a half-life of 6–24 h^42,43^ (Fig. 6b). IF staining for EGFR confirmed the absence of EGFR protein in the TM (Supplementary Fig. 8J, K). Macroscopically, TMs with tissue-specific deletion of *Egfr* did not initially generate the same large tissue mass in response to injury observed in wild-type mice (Figs. 6c, 1b). The day 3 timepoint showed no gross changes from the earlier timepoints. By day 14, when EGFR wild-type TMs had normally grossly repaired, the perforations had failed to close in the EGFR KO mice (Fig. 6c). We examined H&E-stained cross sections of perforated TMs from EGFR KO mice and again observed neither a large build-up of tissue nor closure of the wound by day 7 following perforation (Fig. 6d). In order to determine if the EGFR KO mice had a proliferative defect following wounding, we administered EdU 2 h prior to harvesting of the TM. Whereas wild-type EGFR+ TMs showed a robust proliferative response to injury, EGFR KO TMs showed significant reductions in the number of EdU-labeled cells, particularly on days 1 and 3 (Fig. 6e). Thus, the initiation of the injury response and tissue mass accumulation appear contingent on the presence of EGFR. Evolutionarily, *Areg* appears to be fairly conserved across mammals (Supplementary Fig. 8l), suggesting a potential role for EGFR signaling via Areg activation in other mammalian species. We conclude that *Gpr15L*+ *Areg* + KCs activate EGFR signaling likely in an autocrine or paracrine fashion, driving the initiation of the regeneration program of the TM (Fig. 6f).

## Discussion

The data presented here serves to define and provide a characterization of adult mammalian tympanic membrane regeneration under physiological circumstances without genetic or pharmacologic manipulation. We provide a computational and molecular characterization of epimorphic regeneration of the tympanic membrane after it has suffered a full-thickness injury while maintaining suspension in air. We performed single-cell RNA sequencing to provide an unbiased and comprehensive roadmap of this multi-tissue regenerative process. We highlight the populations that have previously been described to exist within the TM at homeostasis^14^ and show how these populations are transcriptionally altered throughout regeneration. Frumm et al. previously reported that there are two regions of the TM where the stem cell and committed progenitor cells arise – over the malleus and at the pars tensa/pars flaccida junction. We highlight the activation of cells in these same regions in response to injury and the proliferation and migration from these zones to the site of injury, in addition to potentially from the annulus. Moreover, our results show that there are novel cell populations that arise during regeneration, highlighting the ability of the stem cell population to rapidly give rise to a new variant of a TM keratinocyte and providing us important insight into developing candidate molecular signals that drive regeneration.

Herein we characterize the repair of the adult murine TM and present data supporting our postulation that wound healing of this tissue occurs by epimorphic regeneration. Epimorphic regeneration is perhaps best-described in the axolotl limb^44,45^ and is defined by: (1) induction of a unique wounded cell population, (2) creation of a multilineage blastema, and (3) scar-free resolution of injuries. These features are typical of systems in vertebrates more classically defined as having the capacity for epimorphic regeneration^46^. The gross and microscopic histological phenomenology that we have described in this work appears to occur similarly in all other mammals previously studied including humans^47^, rats^48^, chinchillas, dogs, and guinea pigs^18^. This suggests that this regenerative response is a general phenomenon of mammals, rather than being a characteristic of an individual mammalian species.

TM perforations are distinct from other epidermal wounds because they are wounds that injure an epidermal, mesenchymal, and mucosal layer with no anchoring tissue below it (with the notable exception of the rabbit pinna model). Because of the anatomic position of the TM, no underlying tissue scaffold exists to guide regeneration, so the three layers must repair while being suspended in air- very different than the healing of skin tissue, for example, that has many tissues anchored beneath it. Prior histological studies of the healing TM and the data shown here indicate that keratinocytes seem to be the first cell type responding to tissue injury^35^. This is again distinct from other epidermal wounds, where the first steps in repair require coagulation and formation of granulation tissue (containing macrophages, fibroblasts, and extracellular matrix (ECM) components^49^), which is followed by and thought to be a prerequisite for effective epithelialization^49,50^. Moreover, prior work has shown that in epithelial wound healing there is a robust activation of p53, resulting in p21 up-regulation in the wound site keratinocytes which leads the re-epithelialization process^51^. With single-cell RNA sequencing of TMs throughout a regeneration time course, we confirmed that the earliest transcriptional shifts do indeed occur in the keratinocytes. However, the p53 signature observed in other epithelial tissues after injury is not observed in the TM. Furthermore, we observed that there is a unique population of keratinocytes that emerges only after tissue injury, which we have termed the “wounded epithelium”, which does not begin at the wound edge. Based on its transcriptional signature, this population does not appear to be just a differentiated form of a pre-existing stem cell population, which is remarkably similar to what has been documented in single-cell data of the initial wound response in the axolotl^4^. Mammalian skin epithelial stem cells have also been shown to display remarkable plasticity; however, the immediate response to injury comes from basal progenitor cells residing at the wound edge^16^. The TM has its stem cells limited to the malleus and pars tensa/pars flaccida junction, so the initial wound response appears to be acting at a distance in the entire organ. The nature of the signal sent from the injury to the initially responding cells has yet to be elucidated.

Epimorphic regeneration in other vertebrates relies on the wounded epidermis to induce multiple lineages of cells to de-differentiate and form a blastema which continues the process of faithfully regenerating the injured tissue^52–55^. Through our lineage-tracing experiments (Fig. 4a, b), we outlined the migration of cells from the known proliferative centers of the TM^14^ to the perforation site. However it should be noted that our model system selects for cells at the proliferative centers of the pars tensa and pars flaccida junction as well as over the malleus, so this model does not inform what contribution non-proliferating keratinocytes throughout the surface of the tympanic membrane play. At day 1 post-injury, computationally and biologically, we validated the appearance of an undifferentiated transient keratinocyte population that only exists on the TM during regeneration, dissipating by day 14 (Fig. 4g). This proposed migration pattern and emergence suggest blastema-driven regeneration in the TM like that of other non-mammals. The volumetric tissue response seen within the TM (Figs. 1b, 2b) coupled with our data that highlights a new wounded epidermis (Fig. 4) shows a similarity to the wounded epidermis and resulting blastemal bud seen in axolotl regeneration^56^.

Another tenet of epimorphic regeneration is level-specific replacement of tissue. We show here that the epidermal and mesenchymal cell populations undergo transcriptional shifts in regeneration of the TM (Fig. 3). How the different layers communicate in TM regeneration is an area for future investigation, particularly within the multi-lineage blastema containing mesenchymal and immune cells.

A key aspect of epimorphic regeneration is scar-free repair. In general, mammalian tissues are understood to have an extremely limited capacity to regenerate faithfully^57^. At the embryonic stage, mammals have the capacity for scar-free healing and regeneration, but this is quickly lost upon maturation^58,59^. Here, we demonstrate that the murine TM undergoes scar-free repair, as evidenced by the restoration of the organization of collagen fibers in the healed TM (Fig. 2g). This is very unlike what is seen in other reportedly regenerative organs in the adult mammal, like the liver and skin, in which function restores, but the tissues grossly retain a remnant of the injury^16,60^. Not only does the TM display scar-free repair, but it performs this mechanism under the circumstances of a full-thickness wound and while being suspended in air. Thus, understanding TM regeneration may predict important applications in the field of epithelial pathologic scarring.

Recent reports demonstrated in mice that scar-free repair of skin may be possible by mechanical, genetic, or pharmacological manipulations that prevent fibrogenic *Engrailed-*1 positive fibroblasts from emerging during healing^61^. The work we are reporting here complements and extends that work to demonstrate a tissue where epimorphic regeneration is possible under physiological circumstances even without these manipulations. Indeed, based on our scRNA-seq data we did not detect *Engrailed-1* expression in TM fibroblasts throughout the injury time-course (data not shown). It remains to be seen if circumstances that lead to chronic perforation in humans show scarring and activation of this population.

Lastly, we identified the ligand of EGFR, *Areg*, as having a large transcriptional increase in the early stages of TM regeneration with levels restoring back to homeostatic levels by day 14 (Fig. 5b). Interestingly, *Areg* has been shown to be rapidly induced in the wound epidermis of the axolotl post-limb amputation^62^. However, in the axolotl limb, *Areg* expression quickly subsides in normal regeneration, and if *Areg* is aberrantly over-expressed, the limb actually does not regenerate properly, exhibiting epithelial thickening and impaired internal proliferation^62^. Thus, our findings of a large spike and then a rapid decrease in *Areg* expression in the post-perforation TM directly align with other regenerative processes. We were able to genetically modulate EGFR signaling via a conditional knockout mouse where EGFR was deleted from keratinocytes – this resulted in a complete dissipation of the rapid, robust proliferative response to injury we normally see on the TM as well as an inability for TMs to close the injury at all, which indicates that Egfr activation is necessary for the regeneration of the TM.

Taken together, this work suggests that the potential for epimorphic regeneration may be present broadly in adult mammals. Our results show that there are novel cell populations that arise during regeneration, providing us important insight into what molecular signals drive regeneration. We anticipate that a deeper understanding of the process of epimorphic regeneration in the adult mammal TM will permit us to better uncover the critical roadblocks to regeneration in clinical TM conditions, such as chronic TM perforation, as well as in other mammalian tissues. This may ultimately bring us closer to driving whole tissue and organ regeneration in diverse tissues in humans.

## Methods

### Animals

Mice husbandry and procedures were conducted under and approved protocol by the Institutional Animal Care and Use Committee at the University of California, San Francisco (protocol number #192822). Experiments using wild-type mice were performed in strain FVB/NJ. *K5-CreERT2, Egfr*^*fl/fl*^^63^*, R26R-Confetti*^64^, and *mTmG*^41^ mouse lines were acquired from the Jackson Laboratory and maintained on a C57BL/6 background. To generate *Egfr*^*fl/fl*^*; R26*^*mTmG/mTmG*^ conditional KO mice, *Egfr*^*fl/fl*^ mice carrying loxP sites flanking exon 3 of *Egfr* were initially crossed with *R26*^*mTmG/mTmG*^ mice, and then *Egfr*^*fl/fl*^*; R26*^*mTmG/mTmG*^ were crossed with Keratin5-CreER transgenic mice. For most experiments, adult animals—both male and female—between 6 and 12 weeks of age were used. When samples were collected, mice were euthanized using CO_2_ and thoracotomy, and their vasculature was perfused with RNAse-free PBS followed by 10% normal buffered formalin (NBP; sections) or 4% PFA (whole-mounts) to fix the tissue in situ. Whole-mount TMs were either dissected en bloc, or the auditory bulla was isolated and the TM dissected out following fixation and decalcification.

### Perforations

Perforations in mouse TMs were created as previously described^14^. In brief, animals were anesthetized with isoflurane, and the left TM was visualized under a dissection microscope. Perforations were then created in the left anterior pars tensa, using a 25-gauge needle. The right TM of each mouse was kept uninjured, serving as a control.

### TM dissociations

TMs were dissected from 10 6-week-old FVB/NJ mice (all female) per timepoint (1, 3, 7, and 14 days post-injury), with the contralateral ear to the site of perforation serving as an unwounded control. TMs were incubated in dispase (Corning) at 37 °C for ten minutes and then mechanically separated into epidermal and fibrous/mucosal fractions. The epidermal tissue was dissociated in TrypLE (Life Technologies), and the fibrous/mucosal tissue was dissociated in 0.2 mg/mL collagenase P (Sigma-Aldrich) and 5 μg/mL DNAse. Both dissociations were done at 37 °C for 10 min, with trituration every five minutes. Cells were passed through 40 μm strainers, collected by centrifugation, and subjected to removal of dead cells (Miltenyi Biotec). The cells were resuspended at 1000 cells/μL in 0.04% BSA in phosphate buffered saline (PBS), and 30,000 cells were loaded for single cell capture.

### scRNA-seq analysis

Isolated cells were run on the Chromium Controller (10× Genomics) with the Single Cell 3’ Reagent Kit v2, and the generated libraries were sequenced on an Illumina HiSeq 4000. Mouse data was aligned to mm10. Data was run through CellRanger 2.0.0 (10× Genomics) and then analyzed via R primarily through single cell analysis package Seurat version 4^31,65^. In order to limit non-biological sources of variation, standard processing steps were conducted to remove cells expressing less than 200 genes and genes expressed in less than three cells. The data matrices were then log-normalized in a sparse data matrix. Principal component analysis was performed, and the first 10 components that emerged were used to cluster the cells via Seurat-implemented Louvain clustering. We implemented dimensionality reduction analysis and unbiased clustering of cell populations based on similar gene expression patterns without using prior knowledge of population markers to drive the clustering. UMAP plots were generated to create a 2D representation of this multidimensional data. The keratinocytes were analyzed both with and without a linear regression based on cell cycle analysis and assignment of each cell to either S, G1 or G2 phase; no considerable differences were seen between these two analysis modalities, so the data representation used was that of pre-cell cycle regression analysis. Samples from all timepoints following TM injury were harvested and run together in the same batch, so no regression due to batch effects was necessary. All of the individual timepoint read count matrices were combined together and re-clustered via Seurat. This Seurat object was then used for isolating the total keratinocyte, mucosal, immune, and mesenchymal datasets. Individual representations of scVelo plots, Violin plots, heatmaps and subset UMAP plots were adapted from Seurat literature by SMS. If access to specific lines of code wanted, can contact SMS (Sonia.scaria@ucsf.edu).

### Immunofluorescence (IF)

Whole-mount TMs were dissected en bloc, fixed in 4% paraformaldehyde (PFA) at 4 °C for four hours, and decalcified in 5% EDTA overnight. For antibody staining, TMs were permeabilized in PBS with 0.5% Triton X-100 for two hours at room temperature (RT), blocked in PBS with 0.5% Triton X-100 and 10% fetal bovine serum (FBS; blocking buffer) for two hours at RT, and incubated in primary antibody diluted in blocking buffer at 4 °C overnight. TMs were then washed with PBS, incubated in secondary antibodies in blocking buffer for one hour at RT, and again washed with PBS. For addition of a nuclear stain, TMs were incubated in Hoechst dye diluted 1:1000 in PBS for 30 min and washed in PBS. TMs were mounted with Prolong Gold Antifade Mountant (Life Technologies) and sealed with coverslips. Primary antibodies used were rabbit anti-Keratin5 (1:1000) (BioLegend #905501), rabbit anti-Keratin23 (1:100) (LS-Bio #LS-C400571), rabbit anti-Filaggrin (1:100) (LS-Bio #LS-B13455), mouse anti-Collagen II (1:200) (Invitrogen #MA5-12789), rabbit anti-Collagen I (1:100)(Novus #NB600-408), rabbit anti-Areg (1:100) (Invitrogen #PA5-109404), rabbit anti-EGFR (1:100)(Abcam #ab52894) and rabbit anti-pEGFR (1:350) (CST #2234). Secondary antibodies used in these studies were all Alexa fluor-conjugated antibodies (1:250) (Thermo Fisher Scientific).

To prepare paraffin sections of the TM, auditory bullae were fixed with 4% PFA overnight and decalcified in 5% EDTA for three days with daily solution changes. They were then dehydrated, embedded in paraffin, and sectioned at 7- μm thickness. To stain the sections, the paraffin was removed in Histo-Clear II (Electron Microscopy Sciences). The tissue was then rehydrated, and slides were sub-boiled in citrate buffer (10 mM citric acid, 2 mM EDTA, 0.05% Tween-20, pH 6.2) for ten minutes on a heating plate to retrieve the antigens. Samples were permeabilized and blocked at RT for one hour in Animal-Free Blocker (Vector Labs SP-5030) mixed with 0.5% Triton X-100 and 2% normal goat serum (Cell Signaling Technology). The slides were stained with primary and secondary antibodies as described above, but nuclear staining with Hoechst dye was performed at 1:500 for 5 min at RT.

### Imaging

Fluorescent imaging of whole-mount TMs was performed on a Nikon A1R HD confocal microscope with a DU4G filter-based detector, using a Plan Apo Lambda 10× 0.45NA or Super Plan Fluor LWD 20x 0.70NA air objective lens with digital zoom of either 1x or 2x. TMs from *K5-CreERT2; R26R-Confetti* mice were acquired using a Nikon AZ100 macro confocal microscope with a DUS spectral detector using a 4x objective and 2x optical zoom as well as a 1× or 2× digital zoom. Both microscopes used NIS-Elements software for acquisition. Whole-mount images are displayed as maximum intensity projections of z-stacks. TM sections were imaged on a Leica DM6 B microscope. FIJI (ImageJ) software^68^ was used to analyze images, place scale bars, export individual TIFFs, and adjust levels for each channel as needed to maximize image clarity. Imaging of gross anatomy of TMs was performed using a Leica M205 FA stereo microscope and a 2× air objective with the LAS X software.

### RNAscope

Mice were euthanized with CO_2_ and thoracotomy and perfused with RNAse-free PBS followed by 4% paraformaldehyde (PFA) diluted with RNAse-free PBS (for isolation of wholemount TMs) or 10% normal buffered formalin (NBF)(for preparation of tissue sections). In preparation for RNAscope in tissue sections, auditory bullae were isolated, incubated in NBF at RT for 24 h, decalcified, embedded in paraffin, and sectioned at 7-μm thickness. The RNAscope Multiplex Fluorescent Reagent Kit v2 (Advanced Cell Diagnostics) protocol was adapted with the following conditions: manual target retrieval was performed for 15 min, and digestion with Protease Plus was performed for 30 min.

To perform RNAscope in wholemount TMs, a protocol for staining of whole-mount zebrafish embryos was adapted^66^. The TMs were fixed in 4% PFA diluted in RNAse-free PBS for 6 h at RT. They were then washed in PBT (RNAse-free PBS with 0.1% Tween 20), dehydrated in increasing concentrations of methanol (25, 50, and 75% in PBT), and stored in 100% methanol at −20 °C overnight. The next day, the TMs were rehydrated using decreasing concentrations of methanol (75, 50, and 25% in PBT), incubated in Protease III at RT for 10 min, washed, and incubated with the appropriate probes for hybridization at 50 °C overnight. The TMs were then washed in 0.2x SSCT, fixed in 4% PFA at RT for 10 min, and washed again. The signal was amplified and developed following the protocol for the RNAscope Multiplex Fluorescent Reagent Kit v2 protocol.

### EdU administration, detection and analysis

To label proliferating cells in the TM at the time of tissue harvest, mice were injected while awake 2-h prior to takedown with EdU (Carbosynth Limited) was resuspended at 5 mg/mL in saline and passed through a 0.22 μm filter. Mice were each injected with 1 mg (200 μL) by intraperitoneal (IP) injection. To fully label and track the proliferating cell population in the TM over time, EdU was administered continuously, first by IP injection at the start of the experiment and then via supplementation in the drinking water at a concentration of 0.5 mg/mL with 1% sucrose that had been filtered using a 0.2-μm filter. The water supply was changed every three days with care taken to protect the water bottles from light. TMs were dissected and processed as described for IF. The Click-iT EdU Alexa Fluor 488 or 647 Imaging Kit (ThermoFisher Scientific) was used for EdU detection. For combined EdU and protein detection, the IF protocol was followed after EdU detection, starting from the blocking step. Quantification of EdU-labeled cells was performed using Fiji (ImageJ).

### TUNEL staining

A TUNEL kit (C10617, Life technologies) was used to detect apoptotic cells according to the manufacturer’s protocols. Samples were prepared per paraffin protocol outlined above and then incubated with TUNEL working solution for 1 h and shielded from light at 37 °C. The nuclei were stained by Dapi for 30 min. The specimens were imaged using a Nikon A1R HD confocal microscope with a DU4G filter-based detector, using a Super Plan Fluor LWD 20x 0.70NA air objective lens with digital zoom of 1x.

### Lineage tracing with the R26R-confetti reporter

To perform minimal labeling of TM keratinocytes, *K5-CreERT2;R26R-Confetti* mice were given a single IP injection of 30 mg tamoxifen 3 days prior to perforation. At the specified time-points post-injury, whole-mount TMs were dissected en bloc, fixed in 4% PFA at 4 °C for 4 h shielded from light, and decalcified in 5% EDTA at 4 °C overnight. Prior to mounting, TMs were permeabilized in 0.5% Triton X-100 in PBS at RT for 2 h, and nuclei were stained with Hoechst as above.

### *Egfr* Deletion

To induce *Egfr* deletion in vivo, *K5-CreERT2;Egfr*^*fl/fl*^*; R26R*^*mTmG/mTmG*^ mice received IP injections of tamoxifen (0.1 mg/g body weight) dissolved in corn oil at a concentration of 25 mg/mL for 6 days consecutively. Loss of *Egfr* protein was validated by immunofluorescence for Egfr.

### Statistics

Analyses for scRNA-seq data were done using Seurat functions, and GraphPad Prism was used to analyze all other data. Statistical significance was determined by *t*-test when comparing two groups. All representative wholemount images of IF or RNAscope represent an n of at least 3, and all EdU images represent an n of at least 5.

### Reporting summary

Further information on research design is available in the Nature Research Reporting Summary linked to this article.

### Supplementary information

Supplemental material

Reporting summary

## Data Availability

The accession number for the scRNA-seq data reported in this paper is GEO: GSE196692.

## Code availability

Code used in the analysis of this data has been generally outline above and primarily used Seurat version 4 functions with custom edits necessary for our dataset. Codes and all other data are available from the corresponding author upon reasonable request.

## References

[1] AW S, K M (2018). The blastema and epimorphic regeneration in mammals. Dev. Biol..

[2] T V, T B, V S (2009). The wound healing process: an overview of the cellular and molecular mechanisms. J. Int. Med. Res..

[3] JP B, A K (2008). Comparative aspects of animal regeneration. Annu. Rev. Cell Dev. Biol..

[4] Gerber, T. et al. Single-cell analysis uncovers convergence of cell identities during axolotl limb regeneration. 10.1126/science.aaq0681. 10.1126/science.aaq0681 PMC6669047 30262634

[5] Whited JL, Tabin CJ (2009). Limb regeneration revisited. J. Biol..

[6] Leigh, N. D. et al. Transcriptomic landscape of the blastema niche in regenerating adult axolotl limbs at single-cell resolution. 10.1038/s41467-018-07604-0. 10.1038/s41467-018-07604-0 PMC6279788 30514844

[7] Chalkley DT (1954). A quantitative histological analysis of forelimb regeneration in triturus viridescens. J. Morphol..

[8] Seifert AW, Maden M (2014). New insights into vertebrate skin regeneration. Int. Rev. Cell Mol. Biol..

[9] TR G (2016). Comparative analysis of ear-hole closure identifies epimorphic regeneration as a discrete trait in mammals. Nat. Commun..

[10] K M, WF F, SV B (1986). Cellular contribution from dermis and cartilage to the regenerating limb blastema in axolotls. Dev. Biol..

[11] U K, C L, JS P (2009). Improbable appendages: deer antler renewal as a unique case of mammalian regeneration. Semin. Cell Dev. Biol..

[12] Needham AE (1961). Asexual propagation and regeneration: by M. A. Vorontsova and L. D. Liosner, Pergamon Press, London, 1960. pp. xxiv + 489, 70s. Comp. Biochem. Physiol..

[13] Simkin J, Han M, Yu L, Yan M, Muneoka K (2013). The mouse digit tip: from wound healing to regeneration. Methods Mol. Biol..

[14] SM F (2021). A hierarchy of proliferative and migratory keratinocytes maintains the tympanic membrane. Cell Stem Cell.

[15] Blanpain C, Fuchs E (2009). Epidermal homeostasis: a balancing act of stem cells in the skin. Nat. Rev. Mol. Cell Biol. 2009 103.

[16] KAU G, E F (2017). Skin and its regenerative powers: an alliance between stem cells and their niche. Dev. Cell.

[17] CM C, A N (1999). Phenotypic determination of epithelial appendages: genes, developmental pathways, and evolution. J. Investig. Dermatol. Symp. Proc..

[18] AY W (2014). Animal models of chronic tympanic membrane perforation: a ‘time-out’ to review evidence and standardize design. Int. J. Pediatr. Otorhinolaryngol..

[19] Gao T (2017). Management of traumatic tympanic membrane perforation: a comparative study. Ther. Clin. Risk Manag..

[20] Santa Maria PL, Atlas MD, Ghassemifar R (2007). Chronic tympanic membrane perforation: a better animal model is needed. Wound Repair Regen..

[21] ZC L, ZH L, QP Z (2012). Traumatic tympanic membrane perforations: a study of etiology and factors affecting outcome. Am. J. Otolaryngol..

[22] Hall, M. J., Schwartzman, A., Zhang, J. & Liu, X. Ambulatory surgery data from hospitals and ambulatory surgery centers: United States, 2010. (2010). 28256998

[23] RM R (2013). Clinical practice guideline: tympanostomy tubes in children. Otolaryngol. Head. Neck Surg..

[24] M G, S V-G, YC L (1980). Effect of apical epidermal cap on mitotic cycle and cartilage differentiation in regeneration blastemata in the newt, Notophthalmus viridescens. Dev. Biol..

[25] Aragona M (2017). Defining stem cell dynamics and migration during wound healing in mouse skin epidermis. Nat. Commun..

[26] Gantwerker EA, Hom DB (2011). Skin: histology and physiology of wound healing. Facial Plast. Surg. Clin. North Am..

[27] T M (1990). Identification of a major keratinocyte cell envelope protein, loricrin. Cell.

[28] Lippens S, Denecker G, Ovaer P, Vandenabeele P, Declercq W (2005). Death penalty for keratinocytes: apoptosis versus cornification. Cell Death Differ. 2005 122.

[29] Rodrigues M, Kosaric N, Bonham CA, Gurtner GC (2019). Wound healing: a cellular perspective. Physiol. Rev..

[30] Stenfeldt K, Johansson C, Hellström S (2006). The collagen structure of the tympanic membrane: collagen types I, II, and III in the healthy tympanic membrane, during healing of a perforation, and during infection. Arch. Otolaryngol. Head. Neck Surg..

[31] R S, JA F, D G, AF S, A R (2015). Spatial reconstruction of single-cell gene expression data. Nat. Biotechnol..

[32] Farahani RM, Xaymardan M (2015). Platelet-derived growth factor receptor alpha as a marker of mesenchymal stem cells in development and stem cell biology. Stem Cells Int..

[33] Gil-Yarom N (2017). CD74 is a novel transcription regulator. Proc. Natl Acad. Sci. Usa..

[34] Yu, K. S. et al. Development of the Mouse and Human Cochlea at Single Cell Resolution. *bioRxiv* 739680 10.1101/739680 (2019).

[35] DA C, SM F, O A, AD T (2019). Cellular dynamics in early healing of mouse tympanic membranes. Otol. Neurotol..

[36] Stone RC (2016). Epithelial-mesenchymal transition in tissue repair and fibrosis. Cell Tissue Res..

[37] McDonald TM (2013). Zebrafish keratocyte explant cultures as a wound healing model system: differential gene expression & morphological changes support epithelial–mesenchymal transition. Exp. Cell Res..

[38] Klinge U, Dievernich A, Tolba R, Klosterhalfen B, Davies L (2020). CD68+ macrophages as crucial components of the foreign body reaction demonstrate an unconventional pattern of functional markers quantified by analysis with double fluorescence staining. J. Biomed. Mater. Res. Part B Appl. Biomater..

[39] B S, G C, RJ C (2016). EGF receptor ligands: recent advances. F1000Research.

[40] Stoll SW (2016). Membrane-tethered intracellular domain of amphiregulin promotes keratinocyte proliferation. J. Invest. Dermatol..

[41] Muzumdar MD, Tasic B, Miyamichi K, Li L, Luo L (2007). A global double-fluorescent Cre reporter mouse. genesis.

[42] Ray P (2016). Differential protein stability of EGFR mutants determines responsiveness to tyrosine kinase inhibitors. Oncotarget.

[43] Greig MJ (2015). Effects of activating mutations on EGFR cellular protein turnover and amino acid recycling determined using SILAC mass spectrometry. Int. J. Cell Biol..

[44] JD C (2016). Live imaging of axolotl digit regeneration reveals spatiotemporal choreography of diverse connective tissue progenitor pools. Dev. Cell.

[45] Kragl M (2009). Cells keep a memory of their tissue origin during axolotl limb regeneration. Nat 2009 4607251.

[46] Haas BJ, Whited JL (2017). Advances in decoding axolotl limb regeneration. Trends Genet..

[47] Lou ZC, Tang YM, Yang J (2011). A prospective study evaluating spontaneous healing of aetiology, size and type-different groups of traumatic tympanic membrane perforation. Clin. Otolaryngol..

[48] Yilmaz MS (2021). Histological study of the healing of traumatic tympanic membrane perforation after vivosorb and epifilm application. Ear. Nose. Throat J..

[49] Sorg H, Tilkorn DJ, Hager S, Hauser J, Mirastschijski U (2017). Skin wound healing: an update on the current knowledge and concepts. Eur. Surg. Res..

[50] Rittié L (2016). Cellular mechanisms of skin repair in humans and other mammals. J. Cell Commun. Signal..

[51] Yun S, Greco V (2022). From start to finish-a molecular link in wound repair. Sci. (80−)..

[52] LJ C, CM C (2008). Wound epidermis formation and function in urodele amphibian limb regeneration. Cell. Mol. Life Sci..

[53] CS T (1957). The effect of apical cap removal on limb regeneration in Amblystoma larvae. J. Exp. Zool..

[54] AL M (1976). Effects on adult newt limb regeneration of partial and complete skin flaps over the amputation surface. J. Exp. Zool..

[55] Stocum DL (2017). Mechanisms of urodele limb regeneration. Regeneration.

[56] AY W, JL W (2020). Parallels between wound healing, epimorphic regeneration and solid tumors. Development.

[57] Xia H (2018). Tissue repair and regeneration with endogenous stem cells. Nat. Rev. Mater. 2018 37.

[58] Porrello ER (2011). Transient regenerative potential of the neonatal mouse heart. Sci. (80−)..

[59] Tsonis PA, Fox TP (2009). Regeneration according to spallanzani. Dev. Dyn..

[60] Stanger BZ (2015). Cellular homeostasis and repair in the mammalian liver. Annu. Rev. Physiol..

[61] S M (2021). Preventing Engrailed-1 activation in fibroblasts yields wound regeneration without scarring. Science.

[62] Bryant DM (2017). Identification of regenerative roadblocks via repeat deployment of limb regeneration in axolotls. NPJ Regen. Med..

[63] TC L, DW T (2009). Generation and validation of mice carrying a conditional allele of the epidermal growth factor receptor. Genesis.

[64] Snippert HJ (2010). Intestinal crypt homeostasis results from neutral competition between symmetrically dividing Lgr5 stem cells. Cell.

[65] Y H (2021). Integrated analysis of multimodal single-cell data. Cell.

[66] Gross-Thebing T, Paksa A, Raz E (2014). Simultaneous high-resolution detection of multiple transcripts combined with localization of proteins in whole-mount embryos. BMC Biol. 2016 121.

