# Supplementary material for: Epimorphic regeneration in the mammalian tympanic membrane
Source: NPJ Regen Med. 2023 Oct 18;8:58. doi: 10.1038/s41536-023-00332-0 (PMC10584978; doi:10.1038/s41536-023-00332-0)
Supplement: Supplementary file 1 — Supplemental material [file 41536_2023_332_MOESM1_ESM.pdf]

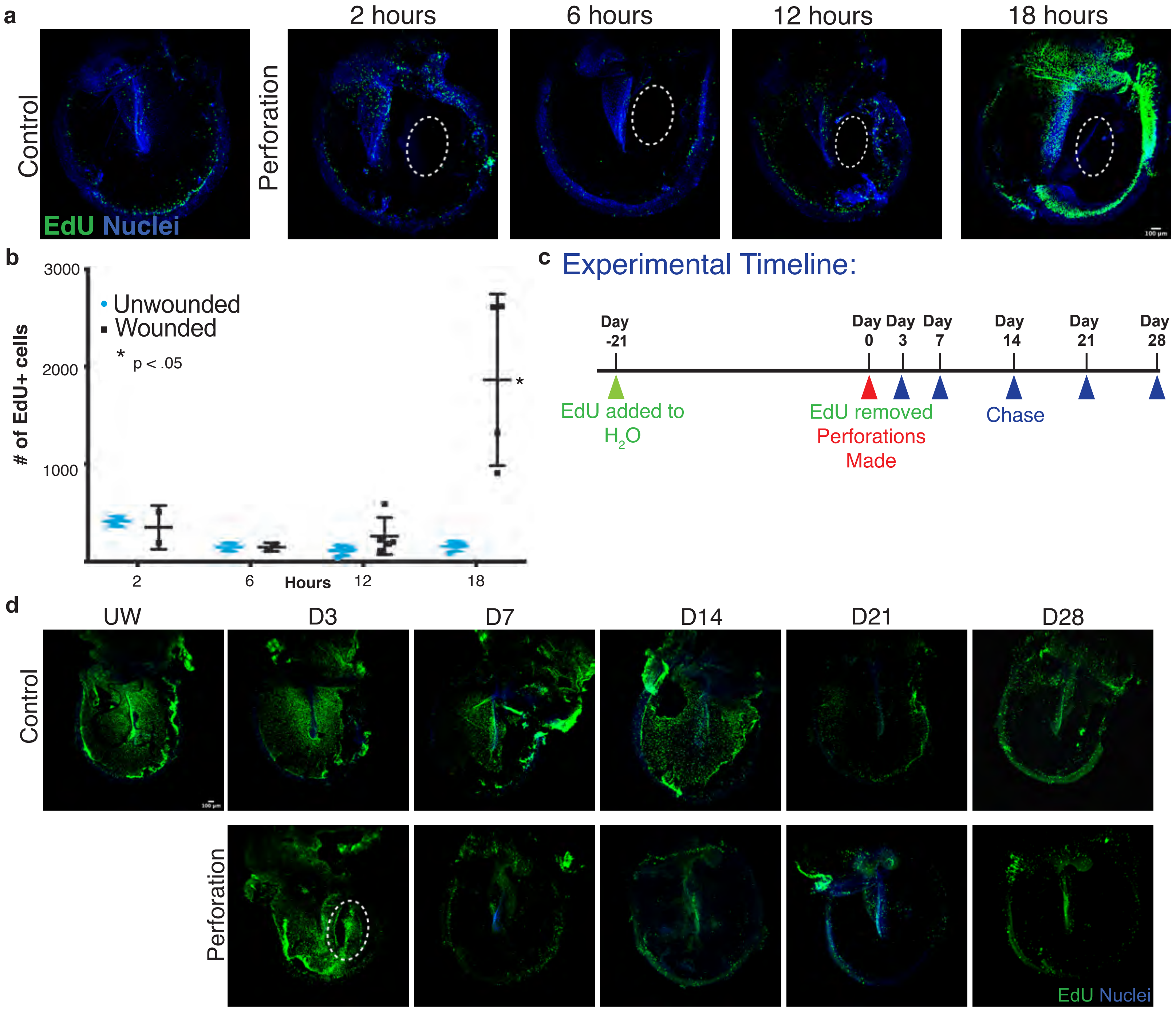
a
2 hours
6 hours
12 hours
18 hours
Control
Perforation
EdU Nuclei
b
# of EdU+ cells
3000
2000
1000
Unwounded
Wounded
* p < .05
2
6
Hours
12
18
c
Experimental Timeline:
Day -21
Day 0
Day 3
Day 7
Day 14
Day 21
Day 28
EdU added to H_2O
EdU removed
Perforations Made
Chase
d
UW
D3
D7
D14
D21
D28
Control
Perforation
EdU Nuclei

Supplementary Figure 1: The TM undergoes a rapid, patterned response to injury. (A) Representative whole-mount TMs from perforations created in left TMs of mice at 2, 6, 12, and 18 hours pre-harvest. EdU was injected IP 2 hours prior to the left (perforated) and right (control) TMs being harvested. The perforation is indicated with a dashed white line. (B) Graph of number of EdU+ cells in a 400 x 1200 µm area over the malleus in response to injury over 18 hours. Results of t-tests for WO vs UW TMs at a single time-point are indicated with the black bars, with the outer limits representing standard deviation; *$p < 0.05$. (C) Timeline describing the pulse-chase experiment. Mice were exposed to EdU continuously for three weeks (pulse), and then the EdU source was removed, the left TMs of the mice were perforated, and the label allowed to dilute for four weeks (chase). (D, E) Representative whole-mount TMs harvested without injury (D) and 3 days, 1 week, 2, 3 and 4 weeks post-injury (E) during the labeling. TMs are stained for EdU (green). Scale bars: 100 µm.

a

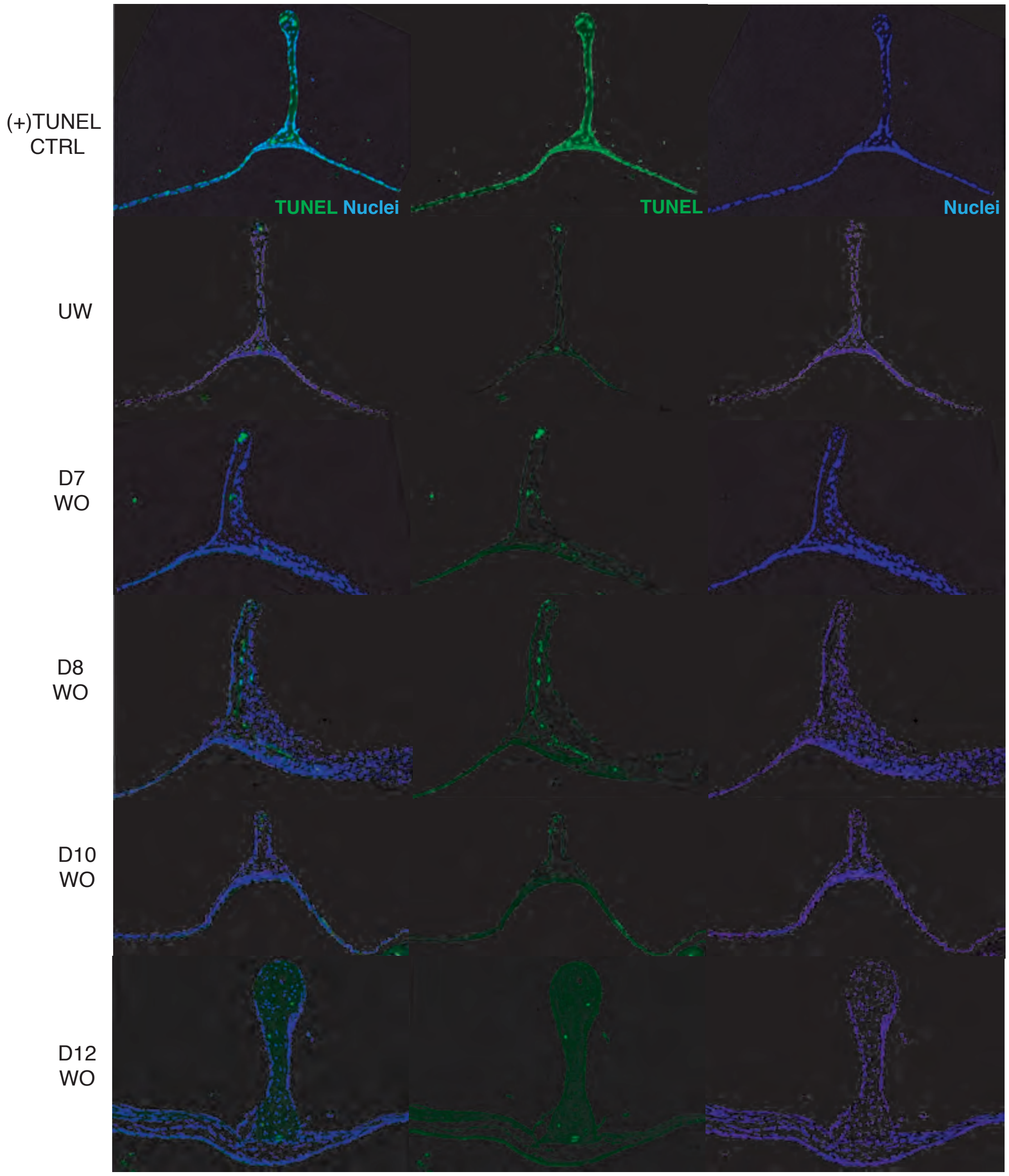
(+)TUNEL CTRL
TUNEL Nuclei
TUNEL
Nuclei
UW
D7 WO
D8 WO
D10 WO
D12 WO

b

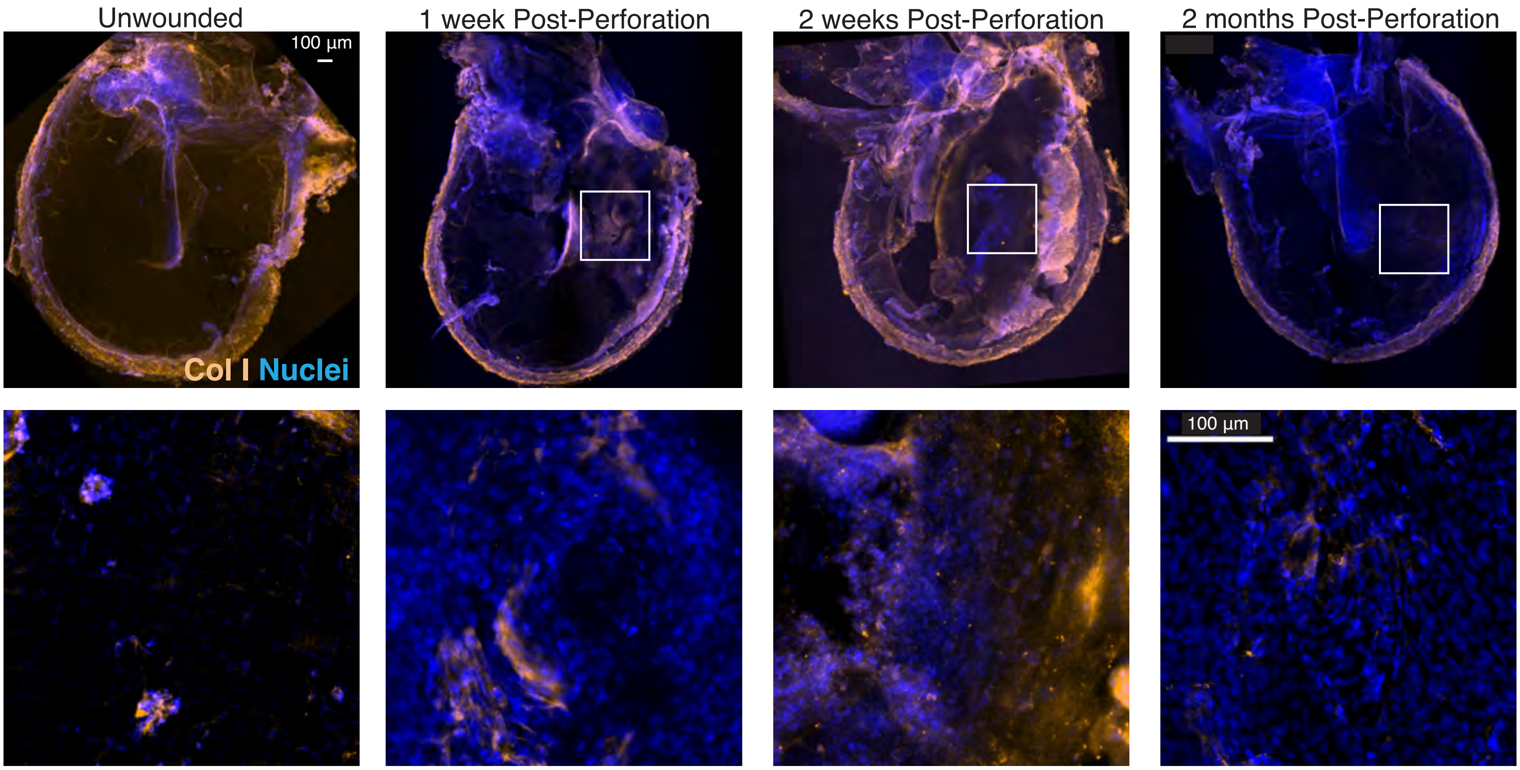
Unwounded
1 week Post-Perforation
2 weeks Post-Perforation
2 months Post-Perforation
100 µm
Col I Nuclei
100 µm

Supplementary Figure 2: Collagen changes in response to wounding dissipate by 2 weeks. (A) TUNEL staining (green) in positive control, unwounded, D7, D8, D10, and D12 TM cross-sections, demonstrating minor apoptotic activity of TM keratinocytes during regeneration. (B)IF for Collagen I (orange) in representative whole-mount TMs harvested at the indicated timepoint post-injury to display the return to wildtype architecture (first panel) in collagen patterning of the TM. Each whole-mount represents at least an n=5. Minor artifactual staining is present at both the unwounded and 2-month timepoints. Scale bars: 100 μm.

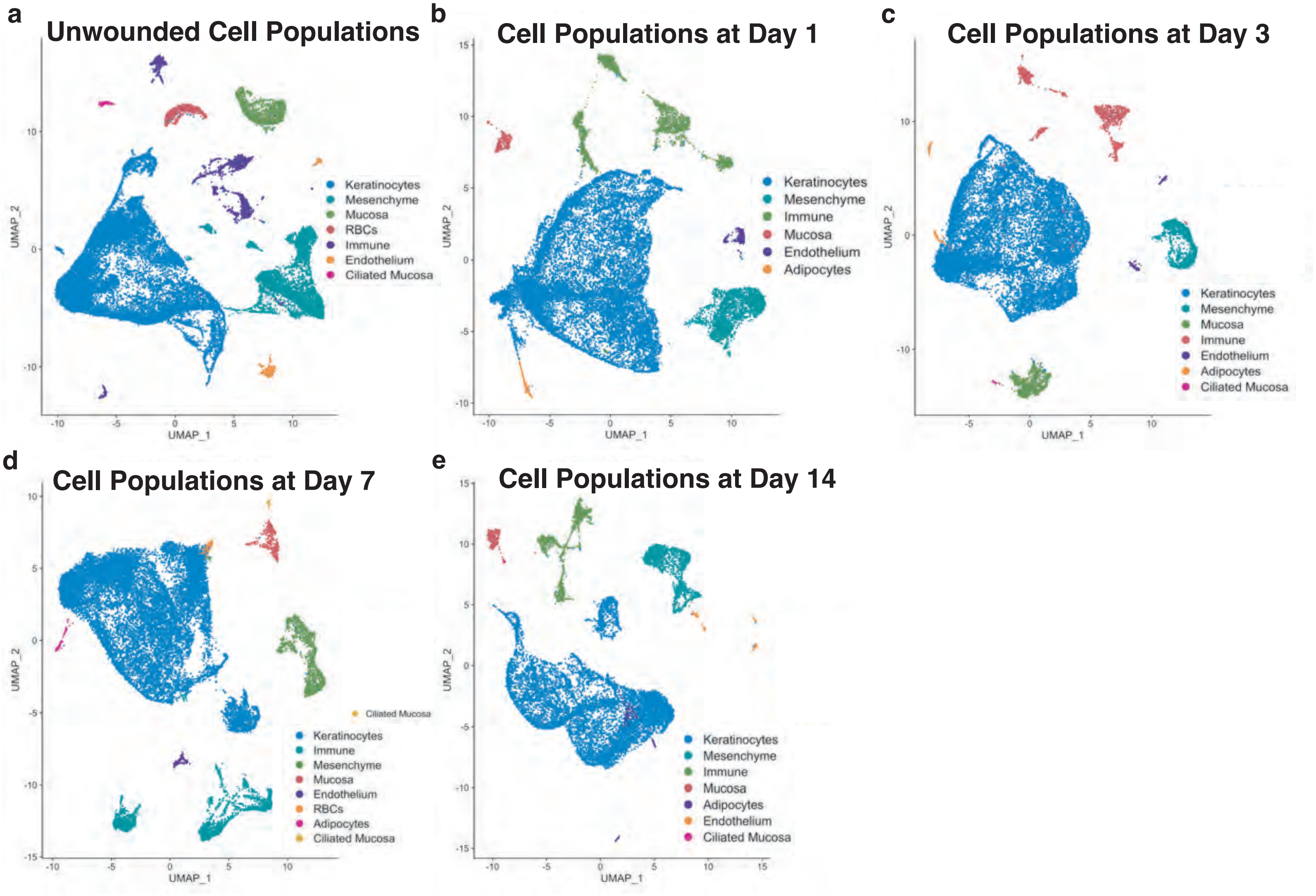

a
Unwounded Cell Populations
Keratinocytes
Mesenchyme
Mucosa
RBCs
Immune
Endothelium
Ciliated Mucosa
UMAP_1
UMAP_2
b
Cell Populations at Day 1
Keratinocytes
Mesenchyme
Immune
Mucosa
Endothelium
Adipocytes
UMAP_1
UMAP_2
c
Cell Populations at Day 3
Keratinocytes
Mesenchyme
Mucosa
Immune
Endothelium
Adipocytes
Ciliated Mucosa
UMAP_1
UMAP_2
d
Cell Populations at Day 7
Ciliated Mucosa
Keratinocytes
Immune
Mesenchyme
Mucosa
Endothelium
RBCs
Adipocytes
Ciliated Mucosa
UMAP_1
UMAP_2
e
Cell Populations at Day 14
Keratinocytes
Mesenchyme
Immune
Mucosa
Adipocytes
Endothelium
Ciliated Mucosa
UMAP_1
UMAP_2

Supplementary Figure 3: ScRNA-seq identifies major populations of cells at each timepoint during the injury response. (A-E) UMAP visualization of all cell clusters in the individual timepoint scRNA-seq data, including the unwounded state (A), day 1(B), day 3 (C), day 7 (D) , and day 14(E), compiled and analyzed by Seurat.

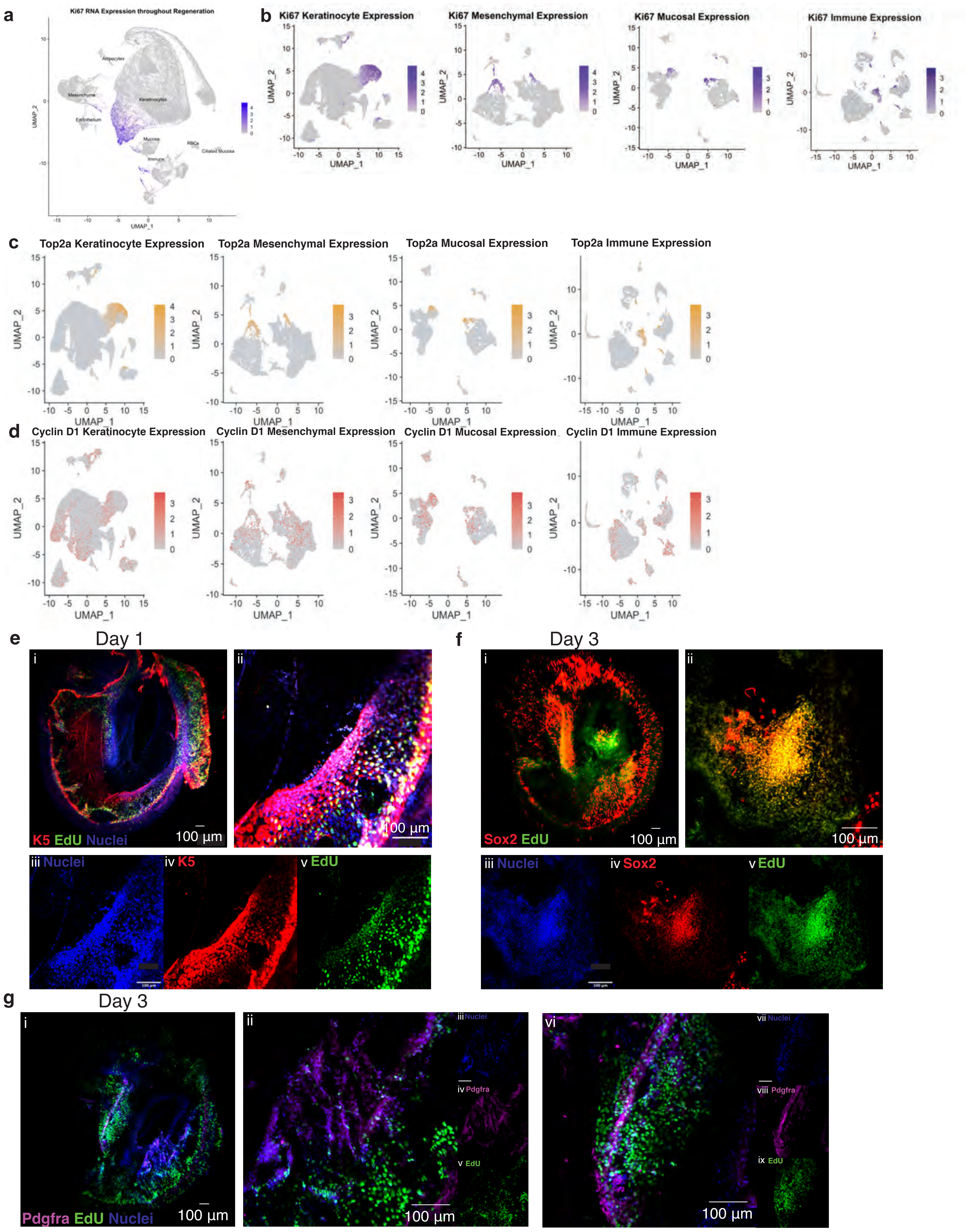
a
Ki67 RNA Expression throughout Regeneration
b
Ki67 Keratinocyte Expression
Ki67 Mesenchymal Expression
Ki67 Mucosal Expression
Ki67 Immune Expression
c
Top2a Keratinocyte Expression
Top2a Mesenchymal Expression
Top2a Mucosal Expression
Top2a Immune Expression
d
Cyclin D1 Keratinocyte Expression
Cyclin D1 Mesenchymal Expression
Cyclin D1 Mucosal Expression
Cyclin D1 Immune Expression
UMAP_1
UMAP_2
e
Day 1
K5 EdU Nuclei
100 µm
iii Nuclei
iv K5
v EdU
f
Day 3
Sox2 EdU
100 µm
iii Nuclei
iv Sox2
v EdU
g
Day 3
Pdgfra EdU Nuclei
100 µm
iii Nuclei
iv Pdgfra
v EdU
vii Nuclei
viii Pdgfra
ix EdU

Supplementary Figure 4: **Markers of proliferation reveal turnover of all layers of the TM**. (A) UMAP plot showing expression of *Mki67* in all the wounded timepoints and unwounded state cells of the murine TM. (B)- (D) UMAP plots showing expression of *Mki67* (B), *Top2a* (C), and *Ccnd1* (D) in the KC fraction, mesenchymal fraction, mucosal fraction, and immune fraction of cells from the total time course. (E) IF for Krt5 (red) co-stained with EdU (Green) in a representative whole-mount TM (i) from one day post-injury. EdU was injected IP 2 hours prior to the TMs being harvested. (ii)-(v) are 4x zoomed in panels of (i). (F) IF for Sox2 (red) co-stained with EdU (Green) in a representative whole-mount TM (i) from 3 days post-injury. (ii)-(v) are 4x zoomed in panels of (i). (G) IF for Pdgfra (pink) co-stained with EdU (Green) in a representative whole-mount TM (i) from three days post-injury. (ii)-(ix) are 4x zoomed in panels of (i). Scale bars: 100 μm.

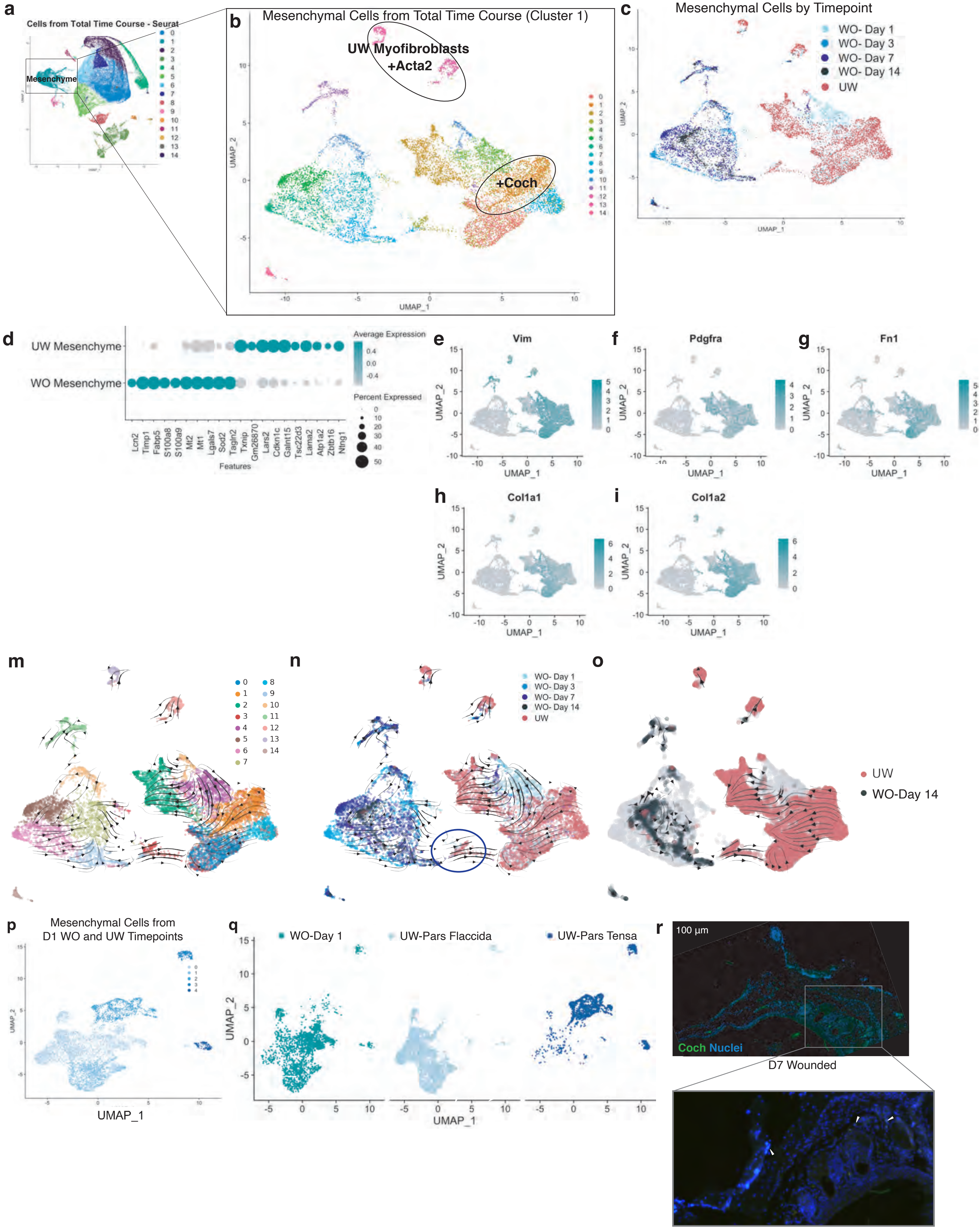
a
Cells from Total Time Course - Seurat
Mesenchyme
b
Mesenchymal Cells from Total Time Course (Cluster 1)
UW Myofibroblasts
+Acta2
+Coch
UMAP_1
UMAP_2
c
Mesenchymal Cells by Timepoint
WO- Day 1
WO- Day 3
WO- Day 7
WO- Day 14
UW
d
UW Mesenchyme
WO Mesenchyme
Average Expression
Percent Expressed
Lcn2
Timp1
Fabp5
S100a8
S100a9
Mt2
Mt1
Lgals7
Sod2
Tagln2
Txnip
Gm26870
Lars2
Cdkn1c
Galnt15
Tsc22d3
Lama2
Atp1a2
Zbtb16
Ntng1
Features
e
Vim
f
Pdgfra
g
Fn1
h
Col1a1
i
Col1a2
m
n
WO- Day 1
WO- Day 3
WO- Day 7
WO- Day 14
UW
o
UW
WO-Day 14
p
Mesenchymal Cells from
D1 WO and UW Timepoints
UMAP_1
q
WO-Day 1
UW-Pars Flaccida
UW-Pars Tensa
UMAP_1
UMAP_2
r
100 µm
Coch Nuclei
D7 Wounded

Supplementary Figure 5: Time-course mesenchymal-specific analysis reveals distinct post-injury transitions. (A) UMAP visualization of the cell clusters identified in scRNA-Seq of all the wounded and unwounded state cells of the murine TM, compiled and analyzed by CellFindR. (B) UMAP visualization of mesenchymal cells from the original clustering, containing the mesenchymal cells from all timepoints, which were re-clustered independent from the other cell types. (C) UMAP visualization of mesenchymal cells, with the cells highlighted based on their original injury timepoint. (D) Dot Plot representation of a subset of the genes that have expression differences in the WO mesenchyme vs the UW mesenchyme. (E-I) Mesenchymal fraction UMAP plots indicating expression of (E) *Vimentin*, (F) *Pdgfra*, (G) *Fibronectin 1,* (H)*Collagen 1a1*, and (I) *Collagen1a2.* (M-O) UMAP visualizations of the mesenchymal populations with RNA velocity vectors super-imposed, calculated using the scVelo package. (M) has cells clustered by their Seurat identities. (N) has cells clustered by timepoint. (O) has only the unwounded and day 14 cells highlighted. Blue circle in (N) highlights vectors moving from the WO to the UW state. (P)UMAP visualization of the Unwounded and Day 1 mesenchymal cells, which were re-clustered independent from the other timepoints. (Q) UMAP visualization of the unwounded pars flaccida, unwounded pars tensa, and day 1 timepoints separated into panels to observe overlapping cells across conditions. (R) RNAscope for *Cochlin* in a TM cross-section from day 7, demonstrating the newly formed multi-lineage blastema of the TM, which includes mesenchymal cells. Scale bar:100 µm.

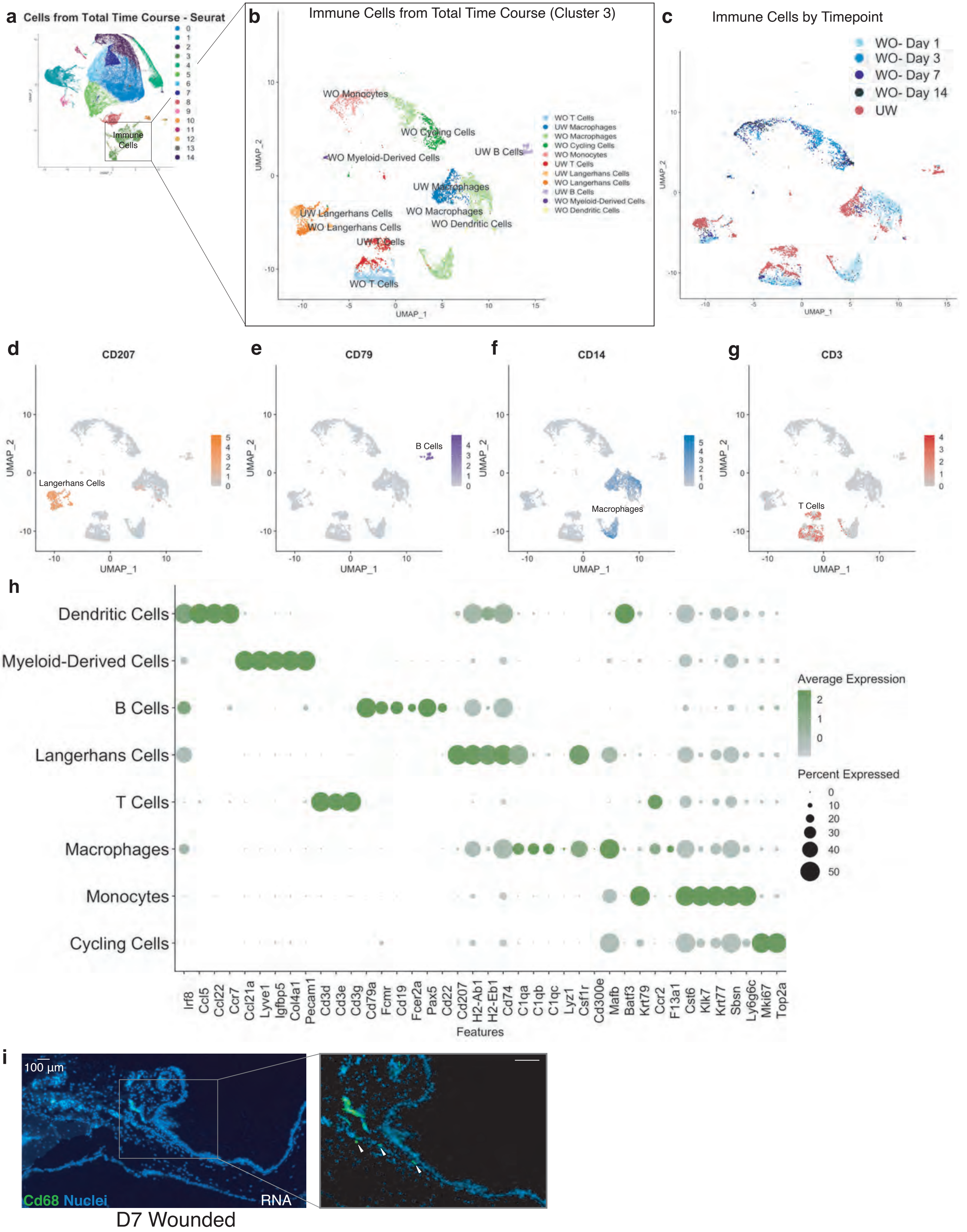
a
Cells from Total Time Course - Seurat
Immune Cells
b
Immune Cells from Total Time Course (Cluster 3)
WO Monocytes
WO Cycling Cells
WO Myeloid-Derived Cells
UW B Cells
UW Macrophages
UW Langerhans Cells
WO Langerhans Cells
WO Macrophages
WO Dendritic Cells
UW T Cells
WO T Cells
WO T Cells
UW Macrophages
WO Macrophages
WO Cycling Cells
WO Monocytes
UW T Cells
UW Langerhans Cells
WO Langerhans Cells
UW B Cells
WO Myeloid-Derived Cells
WO Dendritic Cells
UMAP_1
UMAP_2
c
Immune Cells by Timepoint
WO- Day 1
WO- Day 3
WO- Day 7
WO- Day 14
UW
d
CD207
Langerhans Cells
e
CD79
B Cells
f
CD14
Macrophages
g
CD3
T Cells
h
Dendritic Cells
Myeloid-Derived Cells
B Cells
Langerhans Cells
T Cells
Macrophages
Monocytes
Cycling Cells
Average Expression
Percent Expressed
Irf8 Ccl5 Ccl22 Ccr7 Ccl21a Lyve1 Igfbp5 Col4a1 Pecam1 Cd3d Cd3e Cd3g Cd79a Fcmr Cd19 Fcer2a Pax5 Cd22 Cd207 H2-Ab1 H2-Eb1 Cd74 C1qa C1qb C1qc Lyz1 Csf1r Cd300e Mafb Batf3 Krt79 Ccr2 F13a1 Cst6 Klk7 Krt77 Sbsn Ly6g6c Mki67 Top2a
Features
i
100 µm
Cd68 Nuclei
RNA
D7 Wounded

Supplementary Figure 6: Time-course immune-specific analysis reveals distinct immune populations that arise in response to injury. (A) UMAP visualization of the cell clusters identified in scRNA-Seq of all the wounded and unwounded state cells of the murine TM, compiled and analyzed by CellFindR. (B) UMAP visualization of Cluster 3 from the original clustering, containing the immune cells from all timepoints, which were re-clustered independent from the other cell types. (C) UMAP visualization of immune cells, with the cells highlighted based on their original injury timepoint. (D)- (G) UMAP plots showing expression of (D) *Cd207* to identify Langerhans Cells, (E) *Cd79* to identify B cells, (F) *Cd14* to identify Macrophages, and (G) *Cd3* to identify T cells. (E) Dot Plot visualization of top marker genes for each major immune sub-population present in the TM during regeneration. (I) RNAscope for *Cd68* in a TM cross-section from day 7, demonstrating the newly formed multi-lineage blastema of the TM, which includes immune cells. Scale bar: 100 μm.

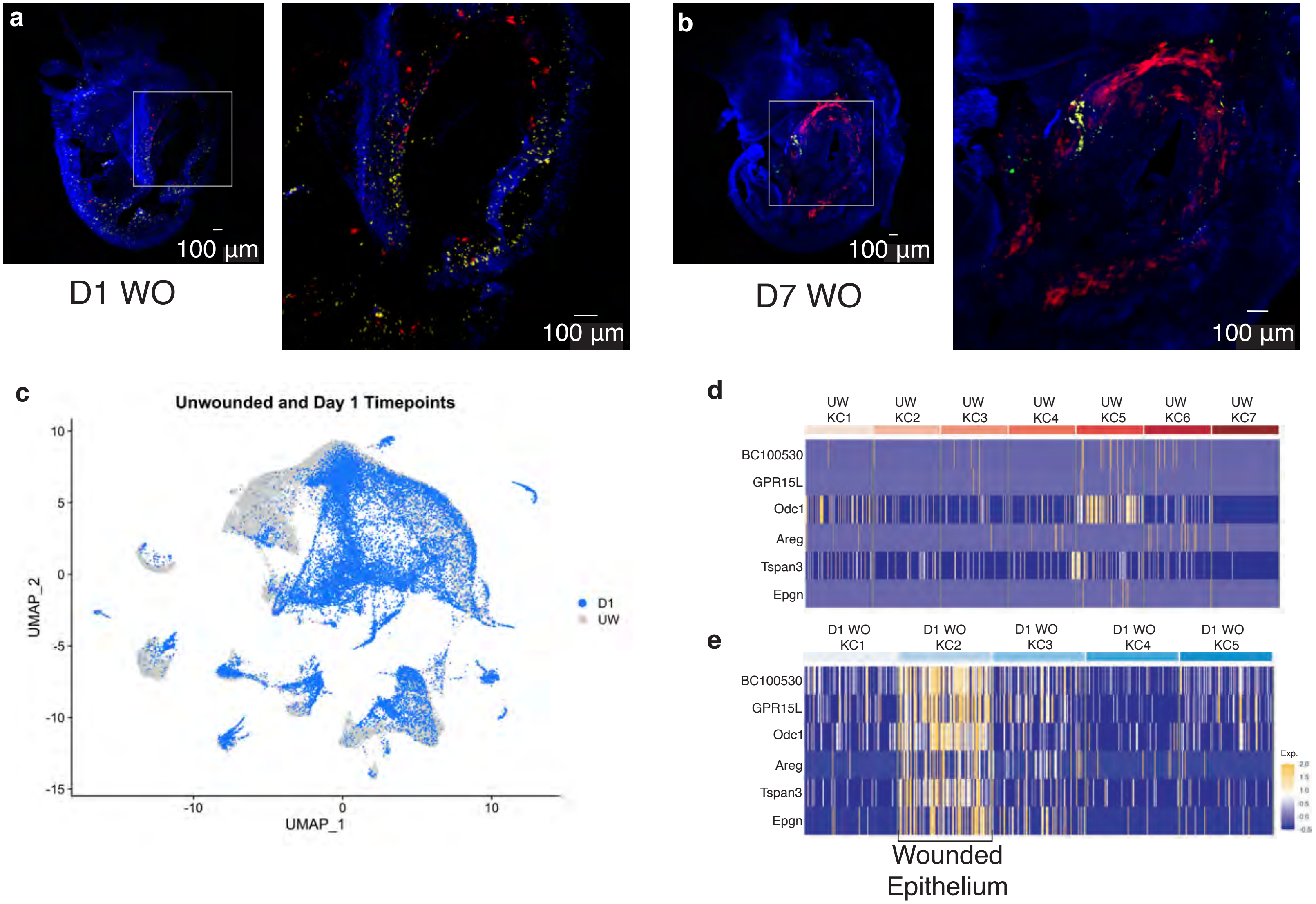

a
100 µm
D1 WO
100 µm
b
100 µm
D7 WO
100 µm
c
Unwounded and Day 1 Timepoints
UMAP_2
UMAP_1
D1
UW
d
UW KC1
UW KC2
UW KC3
UW KC4
UW KC5
UW KC6
UW KC7
BC100530
GPR15L
Odc1
Areg
Tspan3
Epgn
e
D1 WO KC1
D1 WO KC2
D1 WO KC3
D1 WO KC4
D1 WO KC5
BC100530
GPR15L
Odc1
Areg
Tspan3
Epgn
Exp.
Wounded Epithelium

Supplementary Figure 7: Computational analyses of the 1-day post-injury dataset reveal a transcriptional signature for a novel and transient wounded epithelium. (A) Representative whole-mount TM from 1 day post-injury of a Krt5-CreERT2;R26R-Confetti mouse injected with a single dose of 30 mg of tamoxifen 3 days prior to injury to induce minimal labeling of cells. Perforations were created in left TMs of mice on day 0, and the right(control) and left(perforated) TMs were harvested at the indicated timepoint. The injury is outlined with the gray box. Right panel is zoomed in view of the TM corresponding to the location of the box. (B) Representative whole-mount TM from 7 days post-injury from experiment described in (A). Each TM represents an n of at least 5. (C) UMAP visualization of the subset of cells from the Day 1 wounded and unwounded state of cells, re-clustered and colored by timepoint to display the transcriptional shift immediately post-perforation. (D-E) Heat-map showing expression of top genes associated with the wounded epithelium state of the TM in the KC clusters from the unwounded TM (D) and day 1 (E) regenerating TM. Yellow indicates high expression and blue low expression. Each column is a single cell. Scale bar: 100 µm.

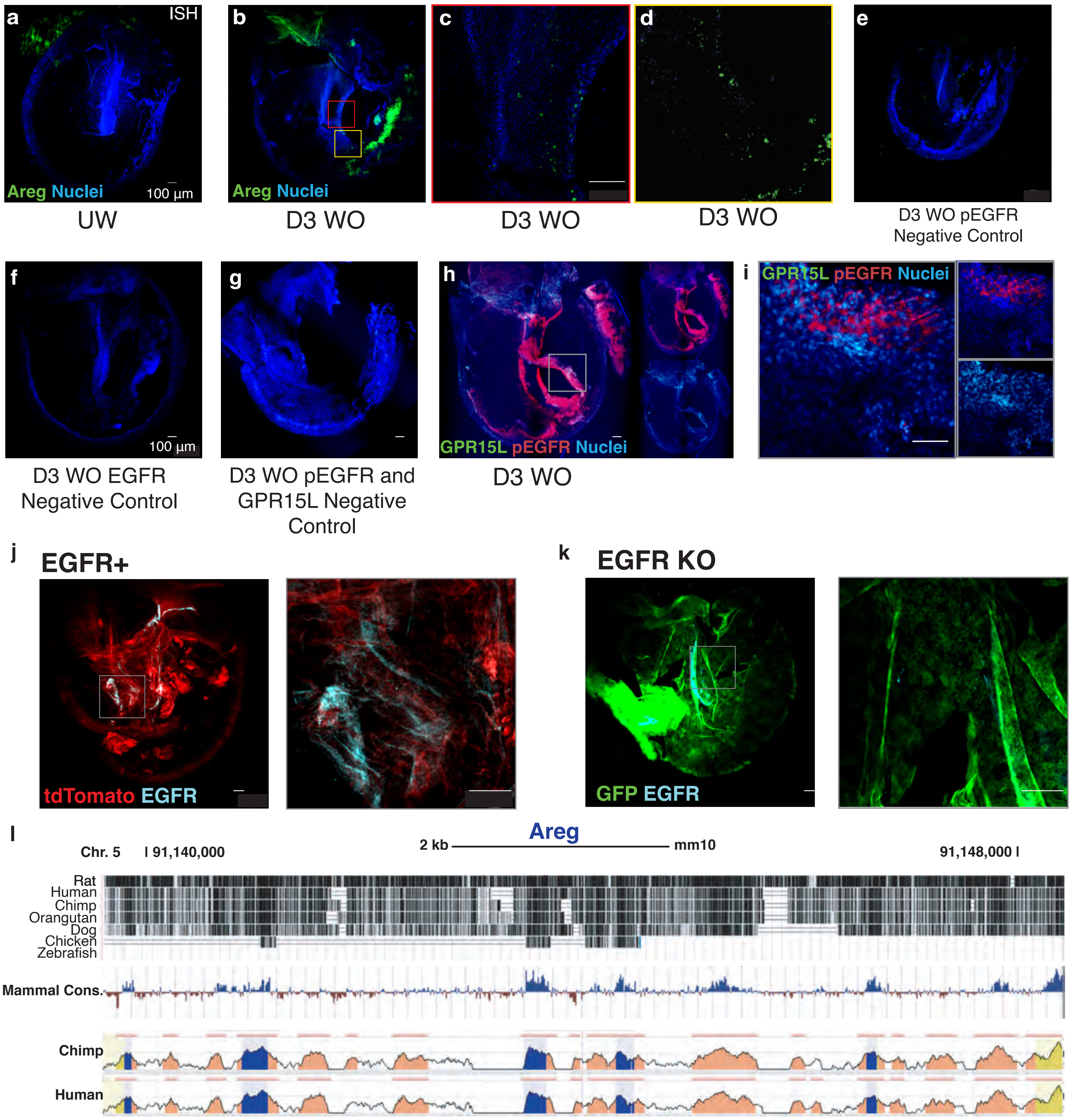
a
ISH
Areg Nuclei
100 µm
UW
b
Areg Nuclei
D3 WO
c
D3 WO
d
D3 WO
e
D3 WO pEGFR Negative Control
f
100 µm
D3 WO EGFR Negative Control
g
D3 WO pEGFR and GPR15L Negative Control
h
GPR15L pEGFR Nuclei
D3 WO
i
GPR15L pEGFR Nuclei
j
EGFR+
tdTomato EGFR
k
EGFR KO
GFP EGFR
l
Areg
Chr. 5
91,140,000
2 kb
mm10
91,148,000
Rat
Human
Chimp
Orangutan
Dog
Chicken
Zebrafish
Mammal Cons.
Chimp
Human

Supplementary Figure 8: Areg RNA expression identifies activated keratinocytes in response to injury. (A-D) RNAscope for *Areg*(green) on representative whole-mount TMs from the unwounded state(A) and day 3 post-injury(B) from wild-type mice. (C-D) represent zoomed in images of the day 3 WO TM corresponding to the gray boxes in (B). (E) Day 3 wounded TM stained with Alexa 488 anti-rabbit secondary to control for artifactual staining for pEGFR experiments. (F) Day 3 wounded TM stained with Alexa 555 anti-rabbit secondary to control for artifactual staining for Egfr experiments. (J-K) IF for Egfr in representative unwounded whole-mount TMs from an $Egfr^{fl/fl}; R26^{mTmG/mTmG}$ ('EGFR+') mouse (J) and a $K5^{Cre\text{-}ERT2/+}; Egfr^{fl/fl}; R26^{mTmG/mTmG}$ ('EGFR KO') mouse (K) injected with tamoxifen. Zoomed in images demonstrate absence of Egfr staining in the KO TM. (L) Mouse *Areg* on chromosome 5 (chr5;mm10). Black bars denote conservation across the listed species; Blue peaks denote areas of high conservation in comparison of the listed species to mouse. Scale bars: 100 μm.
