# Supplementary material for: Epimorphic regeneration in the mammalian tympanic membrane
Source: NPJ Regen Med. 2023 Oct 18;8:58. doi: 10.1038/s41536-023-00332-0 (PMC10584978; doi:10.1038/s41536-023-00332-0)
Supplement: Supplementary file 2 — Reporting summary [file 41536_2023_332_MOESM2_ESM.pdf]

Corresponding author(s): Aaron Tward

Last updated by author(s): 09-12-2023

Reporting Summary

Nature Portfolio wishes to improve the reproducibility of the work that we publish. This form provides structure for consistency and transparency in reporting. For further information on Nature Portfolio policies, see our Editorial Policies and the Editorial Policy Checklist.

Statistics

For all statistical analyses, confirm that the following items are present in the figure legend, table legend, main text, or Methods section.

n/a	Confirmed	
☐	☒	The exact sample size (n) for each experimental group/condition, given as a discrete number and unit of measurement
☐	☒	A statement on whether measurements were taken from distinct samples or whether the same sample was measured repeatedly
☒	☐	The statistical test(s) used AND whether they are one- or two-sided<br>*Only common tests should be described solely by name; describe more complex techniques in the Methods section.*
☒	☐	A description of all covariates tested
☐	☒	A description of any assumptions or corrections, such as tests of normality and adjustment for multiple comparisons
☒	☐	A full description of the statistical parameters including central tendency (e.g. means) or other basic estimates (e.g. regression coefficient) AND variation (e.g. standard deviation) or associated estimates of uncertainty (e.g. confidence intervals)
☒	☐	For null hypothesis testing, the test statistic (e.g. F, t, r) with confidence intervals, effect sizes, degrees of freedom and P value noted<br>*Give P values as exact values whenever suitable.*
☒	☐	For Bayesian analysis, information on the choice of priors and Markov chain Monte Carlo settings
☒	☐	For hierarchical and complex designs, identification of the appropriate level for tests and full reporting of outcomes
☒	☐	Estimates of effect sizes (e.g. Cohen's d, Pearson's r), indicating how they were calculated

Our web collection on statistics for biologists contains articles on many of the points above.

Software and code

Policy information about availability of computer code

Data collection	No code was used in data collection.
Data analysis	Isolated cells were run on the Chromium Controller (10X Genomics) with the Single Cell 3' Reagent Kit v2, and the generated libraries were sequenced on an Illumina HiSeq 4000. Mouse data was aligned to mm10. Data was run through CellRanger 2.0.0 (10x Genomics) and then analyzed via R primarily through single cell analysis package Seurat version 43.

For manuscripts utilizing custom algorithms or software that are central to the research but not yet described in published literature, software must be made available to editors and reviewers. We strongly encourage code deposition in a community repository (e.g. GitHub). See the Nature Portfolio guidelines for submitting code & software for further information.

Data

Policy information about availability of data

All manuscripts must include a data availability statement. This statement should provide the following information, where applicable:

- Accession codes, unique identifiers, or web links for publicly available datasets
- A description of any restrictions on data availability
- For clinical datasets or third party data, please ensure that the statement adheres to our policy

Data Availability
The accession number for the scRNA-seq data reported in this paper is GEO: GSE196692.

Human research participants

Policy information about studies involving human research participants and Sex and Gender in Research.

Reporting on sex and gender	n/a
Population characteristics	*Describe the covariate-relevant population characteristics of the human research participants (e.g. age, genotypic information, past and current diagnosis and treatment categories). If you filled out the behavioural & social sciences study design questions and have nothing to add here, write "See above."*
Recruitment	*Describe how participants were recruited. Outline any potential self-selection bias or other biases that may be present and how these are likely to impact results.*
Ethics oversight	*Identify the organization(s) that approved the study protocol.*

Note that full information on the approval of the study protocol must also be provided in the manuscript.

Field-specific reporting

Please select the one below that is the best fit for your research. If you are not sure, read the appropriate sections before making your selection.

☒ Life sciences ☐ Behavioural & social sciences ☐ Ecological, evolutionary & environmental sciences

For a reference copy of the document with all sections, see nature.com/documents/nr-reporting-summary-flat.pdf

Life sciences study design

All studies must disclose on these points even when the disclosure is negative.

Sample size	Statistical significance was determined by t-test when comparing two groups. All representative wholemount images of IF or RNAscope represent an n of at least 3, and all EdU images represent an n of at least 5.
Data exclusions	n/a
Replication	Each imaging experiment shown in the paper was run multiple times, and the coding was verified with two other coding experts within the lab.
Randomization	Which mouse received perforations and not were randomly selected within gender classes.
Blinding	n/a

Reporting for specific materials, systems and methods

We require information from authors about some types of materials, experimental systems and methods used in many studies. Here, indicate whether each material, system or method listed is relevant to your study. If you are not sure if a list item applies to your research, read the appropriate section before selecting a response.

Materials & experimental systems

n/a	Involved in the study	
☐	☒	Antibodies
☒	☐	Eukaryotic cell lines
☒	☐	Palaeontology and archaeology
☐	☒	Animals and other organisms
☒	☐	Clinical data
☒	☐	Dual use research of concern

Methods

n/a	Involved in the study	
☒	☐	ChIP-seq
☒	☐	Flow cytometry
☒	☐	MRI-based neuroimaging

Antibodies

Antibodies used	Rabbit monoclonal anti-Cytokeratin 10; 1:100 Abcam Cat# ab76318, RRID: AB_1523465<br>Rabbit polyclonal anti-Keratin 5; 1:1000 Biolegend Cat# 905501, RRID: AB_2565050<br>Goat polyclonal anti-Sox2; 1:100 Neuromics Cat# GT15098, RRID: AB_2195800

Rabbit polyclonal anti-Filaggrin; 1:100 LSBio Cat # C293944-100
RRID:AB_2934146

Rabbit polyclonal anti-Keratin 23; 1:20 LSBio Cat# C400571
RRID: n/a
Rabbit polyclonal anti-Collagen II; 1:200 Milllipore Cat# AB2031
RRID: AB_91206

Rabbit polyclonal anti-Collagen I; 1:100 LSBio Cat# LS-B342
RRID: AB_1242263
Rabbit polyclonal anti-Areg; 1:100 Invitrogen Cat# PA5-109404
RRID: AB_2854815
Rabbit monoclonal anti-EGFR; 1:100 Abcam Cat# ab52894
RRID: AB_869579
Rabbit monoclonal anti-phospho-EGFR (Tyr 1068); 1:350
CST Cat# 2234
RRID: AB_2096270
Goat polyclonal anti-PDGFR-alpha; 1:100 R&D Systems Cat# AF1062
RRID: AB_2236897
Goat anti-Rabbit IgG (H+L) Cross-Adsorbed, Alexa Fluor 555; 1:250 Thermo Fisher Scientific Cat# A-21428, RRID: AB_2535849
Goat anti-Rat IgG (H+L) Cross-Adsorbed, Alexa Fluor 488; 1:250 Thermo Fisher Scientific Cat# A-11006, RRID: AB_2534074
Donkey anti-Goat IgG (H+L) Cross-Adsorbed; 1:250 Thermo Fisher Scientific Cat# A-11055, RRID: AB_2534102

Validation

RRIDs listed above for all antibodies

Animals and other research organisms

Policy information about studies involving animals; ARRIVE guidelines recommended for reporting animal research, and Sex and Gender in Research

Laboratory animals

Mouse: FVB/NJ From: The Jackson Laboratory Cat# 001800, RRID: IMSR_JAX:001800
Mouse: Krt5-CreERT2; Tg(KRT5-cre/ERT2)1Blh From: Sarah Knox MGI: 4358332
Mouse: R26R-Confetti; Gt(ROSA)26Sortm1(CAG-Brainbow2.1)Cle/J From: Ophir Klein RRID: IMSR_JAX:013731
Mouse: mT/mG; Gt(ROSA)26Sortm4(ACTB-tdTomato,-EGFP)Luo/J From: Ophir Klein RRID: IMSR_JAX:007676

Wild animals

n/a

Reporting on sex

n/a

Field-collected samples

n/a

Ethics oversight

Mice husbandry and procedures were conducted following the guidelines of the Institutional Animal Care and Use Committee at the University of California, San Francisco (approval number #192822).

Note that full information on the approval of the study protocol must also be provided in the manuscript.
